# Plastome evolution of *Engelhardia* facilitates phylogeny of Juglandaceae

**DOI:** 10.1186/s12870-024-05293-0

**Published:** 2024-07-06

**Authors:** Yue Huang, Xin-Jie Jin, Can-Yu Zhang, Pan Li, Hong-Hu Meng, Yong-Hua Zhang

**Affiliations:** 1 College of Life and Environmental Science, Wenzhou University, Wenzhou, 325035 China; 2 Plant Phylogenetics and Conservation Group, Center for Integrative Conservation & Yunnan Key Laboratory for Conservation of Tropical Rainforests and Asian Elephants, Xishuangbanna Tropical Botanical Garden, Chinese Academy of Sciences, Mengla, 666303 China; 3 Yunnan Normal University, Kunming, 650500 Yunnan China; 4 Laboratory of Systematic & Evolutionary Botany and Biodiversity, College of Life Sciences, Zhejiang University, Hangzhou, 310058 China; 5 Zhejiang Provincial Key Laboratory for Water Environment and Marine Biological Resources Protection, Wenzhou University, Wenzhou, 325035 China

**Keywords:** *Engelhardia*, Plastomes, Phylogenetic relationships, Insertion and deletion, Codon usage pattern

## Abstract

**Background:**

*Engelhardia* (Juglandaceae) is a genus of significant ecological and economic importance, prevalent in the tropics and subtropics of East Asia. Although previous efforts based on multiple molecular markers providing profound insights into species delimitation and phylogeography of *Engelhardia*, the maternal genome evolution and phylogeny of *Engelhardia* in Juglandaceae still need to be comprehensively evaluated. In this study, we sequenced plastomes from 14 samples of eight *Engelhardia* species and the outgroup *Rhoiptelea chiliantha*, and incorporated published data from 36 Juglandaceae and six outgroup species to test phylogenetic resolution. Moreover, comparative analyses of the plastomes were conducted to investigate the plastomes evolution of *Engelhardia* and the whole Juglandaceae family.

**Results:**

The 13 *Engelhardia* plastomes were highly similar in genome size, gene content, and order. They exhibited a typical quadripartite structure, with lengths from 161,069 bp to 162,336 bp. Three mutation hotspot regions (*TrnK-rps16*, *ndhF-rpl32*, and *ycf1*) could be used as effective molecular markers for further phylogenetic analyses and species identification. Insertion and deletion (InDels) may be an important driving factor for the evolution of plastomes in Juglandoideae and Engelhardioideae. A total of ten codons were identified as the optimal codons in Juglandaceae. The mutation pressure mostly contributed to shaping codon usage. Seventy-eight protein-coding genes in Juglandaceae experienced relaxed purifying selection, only *rpl22* and *psaI* genes showed positive selection (*Ka/Ks* > 1). Phylogenetic results fully supported *Engelhardia* as a monophyletic group including two sects and the division of Juglandaceae into three subfamilies. The *Engelhardia* originated in the Late Cretaceous and diversified in the Late Eocene, and Juglandaceae originated in the Early Cretaceous and differentiated in Middle Cretaceous. The phylogeny and divergence times didn’t support rapid radiation occurred in the evolution history of *Engelhardia*.

**Conclusion:**

Our study fully supported the taxonomic treatment of at the section for *Engelhardia* species and three subfamilies for Juglandaceae and confirmed the power of phylogenetic resolution using plastome sequences. Moreover, our results also laid the foundation for further studying the course, tempo and mode of plastome evolution of *Engelhardia* and the whole Juglandaceae family.

**Supplementary Information:**

The online version contains supplementary material available at 10.1186/s12870-024-05293-0.

## Introduction

The walnut family (Juglandaceae) containing ca. 60 extant species belonging to ca. 10 genera, a woody family in the order Fagales, is mainly distributed in subtropical to tropical forests [1–3]. Members of this family play an important role in local forest ecosystems, and some of them are important nut, timber, and medicinal trees. According to APG IV (2016), Juglandaceae is grouped into three subfamilies: Rhoipteleoideae, Engelhardioideae, and Juglandoideae [4]. Among the three subfamilies of Juglandaceae, species of the Juglandoideae subfamily were very common in temperate deciduous forests in the Northern Hemisphere, while species of the Engelhardioideae subfamily and the Rhoipteleoideae subfamily were mainly distributed in subtropical and tropical forests [2].

In the Engelhardioideae subfamily, species of *Engelhardia* Lesch. ex Blumewidely distribute in the tropical and subtropical regions of eastern Asia [5], which are widely used in wood and tea, and also play a significant role in the ecosystem [6, 7]. There are about 9 *Engelhardia* species in China, which mainly occur in the southwest, south to southeast [7]. The *Engelhardia* species are deciduous or semi-evergreen trees or evergreen tree, often with even-pinnate compound leaves, monoecious or dioecious, fruit nut-like, when the fruit is ripe, the bracts grow, membranous, and connate with the fruit to form a nutlet with 3-lobed wings [8]. In previous studies on the species delimitation of *Engelhardia* [7, 9] and the phylogeography of two trees species (i.e., *E. roxburghiana* and *E. fenzelii*) [10], combined plastid regions (*psbA*-*trnH*, *trnL*-*trnF*, *rps16*, *trnS*-*trnG*, and *rpl32*-*trnL*), one nuclear ribosomal internal transcribed spacer (nrITS), and Microsatellite (nSSR) data were used. Yet to date, except in individual cases [3, 11, 12], comparative analyses of multiple *Engelhardia* plastome are still lacking.

Plastomes, as critical organelles, play a pivotal role in plant cells, underscored by their widespread application in evolutionary and phylogenetic studies [13, 14]. Their importance is attributed to maternal uniparental inheritance and a highly conserved structure, making them valuable in dissecting plant evolutionary histories and relationships across a wide array of studies [15, 16]. Plastome is usually a closed circular tetrad structure composed of DNA double-stranded molecules, including a large single copy region (LSC), a small single copy region (SSC) and a pair of inverted repeat region (IRa/IRb) [17]. Early phylogenetic analyses used partial plastomes DNA sequences. However these fragments did not have enough information to distinguish closely related plant species, while whole plastid genomes can provide in-depth information to improve our understanding of species evolution [18]. The complete plastomes had made great progress in elucidating the relationship in monocot [19], and also explained the relationship between several major lineages of angiosperms [20]. Meanwhile, plant plastome gene and genome evolving during the process of speciation can help us understand how species adapts to diverse ecological habitats [14].

In the study of phylogeny and evolution of species, fossils play a crucial role in determining the time of species differentiation [21]. For example, the well preserved Rhynie Chert fossils were helpful to provide insights into the life cycle of early land plants [22], and the usage of fossil correction and molecular clock methods can well support the pre-Cretaceous origin of angiosperms [23]. Previous studies based on fossil evidence have greatly promoted our understanding of Juglandaceae as a whole [2, 24], but for subfamily Engelhardioideae, generally only showing the differentiation time of *E. roxburghiana* due to insufficient sampling. Therefore, the divergence times of the Engelhardioideae species remain unsolved. What is the divergence time within the *Engelhardia*? Are the results based on different fossil calibration points consistent with those of predecessors? It is necessary to increase the species sampling of the *Engelhardia* to explore the phylogenetic relationship, divergence and orgin of Juglandaceae.

In this study, a total of 14 individuals from eight species of *Engelhardia* (*E. anminiana*, *E. fenzelii*, *E. hainanensis*, *E. roxburghiana*, *E. serrata*, *E. spicata*, *E. spicata* var. *rigida*, and *E. villosa*) and one outgroup species *Rhoiptelea chiliantha* were newly sequenced (Table 1), and 42 plastome sequences from 36 Juglandaceae species and six outgroup species were downloaded from GenBank. The whole plastomes were used to explore evolution and the deep phylogenetic relationship among the species of *Engelhardia* and (or) other genera, subfamilies, even the whole Juglandaceae family. Our specific goals were as follows: (1) to compare the plastomes and identify the variation in *Engelhardia*; (2) to identify genomic structural variation across Juglandaceae plastomes; (3) to deepen the understanding on the codon usage bias and gene evolution in Juglandaceae plastomes; (4) to infer and test the phylogenetic relationship and divergence time among the genera and subfamilies of Juglandaceae using plastome data.

**Table 1:** Taxa, voucher and GenBank accession numbers of *Engelhardia* species and *Rhoiptelea chiliantha* sequenced in this study

Species	DNA code	Voucher no	Collector	Plastome	Locality
*Engelhardia anminiana*		MHH2018001-02	Hong-Hu Meng	OR208248	Sulawesi province, Indonesia
*Engelhardia fenzelii*	JNSX01	ZYH19072801	Yong-Hua Zhang	OP480035	Jingning, Lishui, Zhejiang, China
	TTD01	ZYH17102801	Yong-Hua Zhang	OP480037	Tiantangding, Guangzhou, Guangdong, China
*Engelhardia hainanensis*	02	MHH20170514001A	Hong-Hu Meng	OR208247	Bawangling, Changjiang, Hainan, China
	HN01	ZYH18072101	Yong-Hua Zhang	OP480038	Jianfengling, Ledong, Hainan, China
*Engelhardia roxburghiana*	BPZ11	ZYH17120911	Yong-Hua Zhang	OP480042	Baipenzhu, Huizhou, Guangdong, China
	JFL02	ZYH18072103	Yong-Hua Zhang	OP480031	Jianfengling, Ledong, Hainan, China
	TPS06	ZYH17121606	Yong-Hua Zhang	OP480033	Huaping, Guilin, Guangxi, China
	XSBN01	ZYH19011503	Yong-Hua Zhang	OP480034	Xishuangbanna Tropical Botanical Garden, Yunnan, China
*Engelhardia serrata*		MHH201800103-10	Hong-Hu Meng	OR208250	Sulawesi province, Indonesia
*Engelhardia spicata*		MHH2018092101-01	Hong-Hu Meng	OR208253	Xishuangbanna Tropical Botanical Garden, Yunnan, China
*Engelhardia spicata* var.* rigida*		MHH20180922015-16	Hong-Hu Meng	OR208251	Sumatra Island, Indonesia
*Engelhardia villosa*		MHH2018032813-20	Hong-Hu Meng	OR208252	Niukong, Lvchun, Yunnan, China
*Rhoiptelea chiliantha*	MWS2	LP174627	Pan Li	OP480039	Gulinqing, Maguan, Yunnan, China

## Results

### Characteristics of *Engelhardia* plastomes

**Fig. 1:**
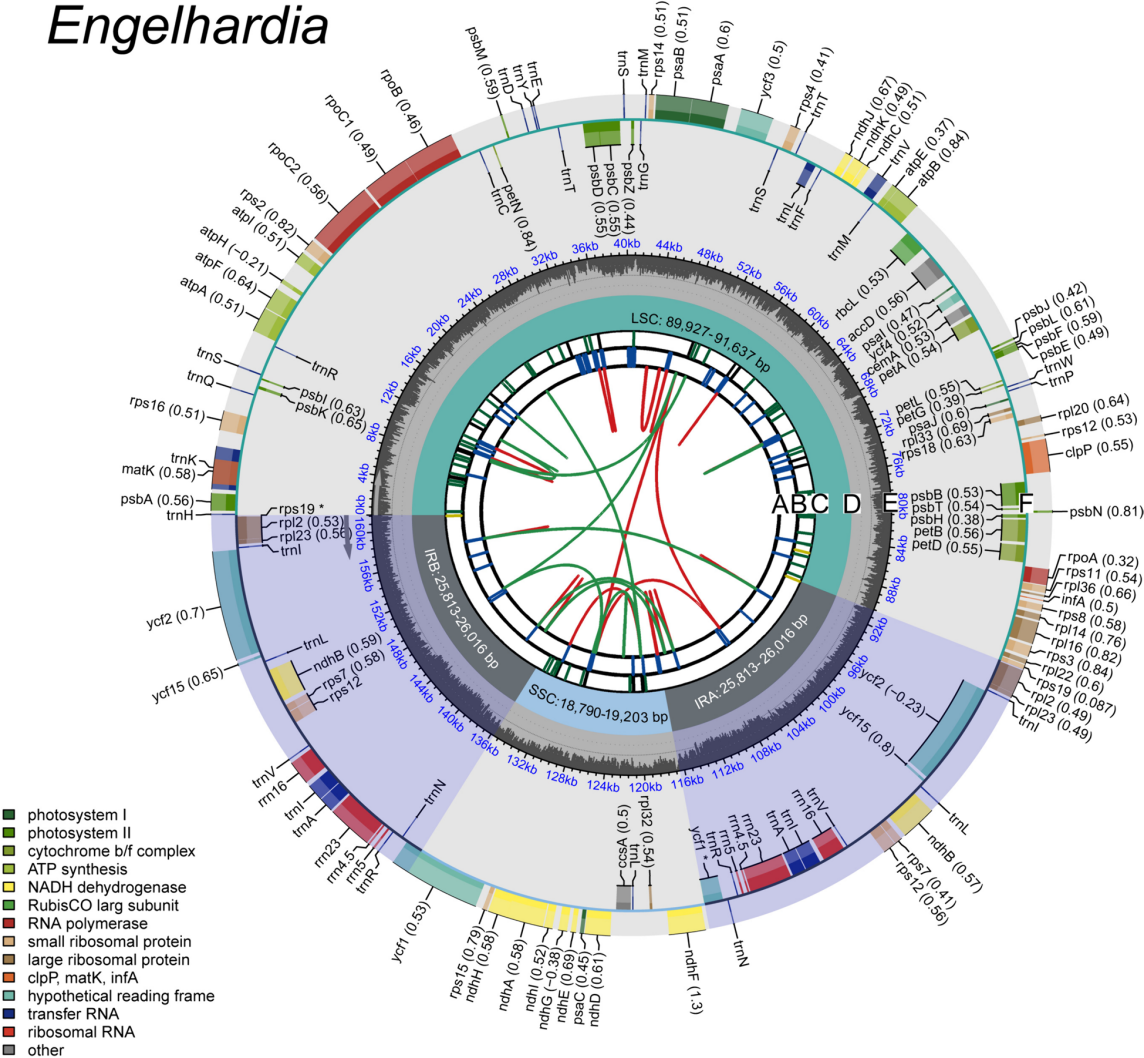
Gene map of *Engelhardia* plastomes. The species name is displayed in the upper left corner, and the genome map includes 5 tracks. From the inside out, the first track (**A**) displays forward and reverse repeats connected by red and green arcs. The second track (**B**) shows the tandem repeats, represented by blue line segments. The third track (**C**) displays the microsatellite sequences, represented by green and yellow line segments. The fourth track (**D**) displays large single copy (LSC), small single copy (SSC), and inverted repeat (IRa and IRb). The fifth track (**E**) displays the GC content of the genome. The genes are distributed in the outermost circle (**F**), the optional codon pusage bias is displayed in parentheses after the gene name. The genes shown inside and outside of the circle are transcribed in clockwise and counterclockwise directions, respectively. Genes from different functional groups are shown in different colors

The lengths of the complete plastomes of *Engelhardia* were slightly different, ranging from 161,069 bp to 162,336 bp, exhibiting a quadripartite structure with a large singlecopy (LSC) region (89,927–91,637 bp), dual inverted repeat (IR) regions (25,813–26,016 bp), and a small single-copy (SSC) region (18,790–19,203 bp) (Fig. 1, Table 2). There were a total of 134 genes were identified in the newly sequenced plastomes, including 88 protein-coding genes (CDS), two pseudogenes (*Ψycf1*、*Ψrps19*), 37 transfer RNA (tRNA) genes and eight ribosomal (rRNA) genes (Table 2). The *ycf1* in the IRb region (*Ψycf1*) and the *rps19* in IRa region (*Ψrps19*) of all *Engelhardia* species were identified as pseudogenes (Table S2)*.* Among these genes, there were 18 intron-containing genes, of which three genes *rps12*, *clpP* and *ycf3*, had two introns, and the rest contained a single intron (*trnA*-*UGC*, *trnG*-*UCC*, *trnI*-*GAU*, *trnK*-*UUU*, *trnL*-*UAA*, *trnV*-*UAC*, *rpl2*, *rpl16*, *rps16*, *rpoC1*, *atpF*, *ndhA*, *ndhB*, *petB*, and *petD*) (Table S2). These newly-generated *Engelhardia* plastomes were deposited in GenBank (Assession Number showed in Table 1).

**Table 2:** Complete features of 13 newly assembled *Engelhardia* plastomes and one *Rhoiptelea chiliantha* plastome

Species	Total (bp)	LSC (bp)	SSC (bp)	IR (bp)	CDS (bp)	Total GC content (%)	Total genes	CDS	Pseudo	tRNA genes	rRNA genes
*E. anminiana*	162,088	91,302	19,054	25,866	78,969	35.9	134	88	2	37	8
*E. fenzelii*_JNSX01	161,069	89,927	19,112	26,015	78,867	36.0	134	88	2	37	8
*E. fenzelii*_TTD01	161,105	89,964	19,111	26,015	78,867	36.0	134	88	2	37	8
*E. hainanensis*_02	161,574	91,157	18,791	25,813	79,137	35.8	134	88	2	37	8
*E. hainanensis*_HN01	161,572	91,156	18,790	25,813	79,137	35.8	134	88	2	37	8
*E. roxburghiana*_BPZ11	161,713	90,478	19,203	26,016	78,906	35.9	134	88	2	37	8
*E. roxburghiana*_JFL02	161,511	90,373	19,106	26,016	78,915	35.9	134	88	2	37	8
*E. roxburghiana*_TPS06	161,713	90,478	19,203	26,016	78,906	35.9	134	88	2	37	8
*E. roxburghiana*_XSBN01	161,667	90,448	19,187	26,016	78,906	35.9	134	88	2	37	8
*E. serrata*	162,161	91,313	19,092	25,878	78,852	35.9	134	88	2	37	8
*E. spicata*	161,551	90,937	18,890	25,862	79,113	35.8	134	88	2	37	8
*E. spicata* var. *rigida*	161,520	90,951	18,871	25,849	79,122	35.9	134	88	2	37	8
*E. villosa*	162,336	91,637	19,043	25,828	79,143	35.8	134	88	2	37	8
*R. chiliantha*_MWS2	161,702	90,447	19,081	26,087	79,182	36.1	133	88	1	37	8

The overall GC content of *Engelhardia* plastomes was 35.8%–36.0% (Table S3), and the GC contents of coding sequence (CDS) regions was 37.2%–37.3%. We found that the GC content of LSC (33.2%–33.6%) and SSC (29.3%–29.6%) region were lower than those of IR regions (42.6%–42.7%) (Table S3).

### Comparative analysis of *Engelhardia* plastomes

Multiple plastomes comparison among all the *Engelhardia* species using mVISTA and Mauve alignment showed high degree of collinearity. It was found that the composition and sequence of genes in *Engelhardia* were highly consistent, and no inversion or translocation of DNA fragment rearrangement was detected in the sequences (Fig. S2). The regions with relatively low identity were *rps16*_*trnQ*-*UUG*, *trnS*-*GCU*_*trnG*-*UCC*, *trnT*-*GGU*_*psbD*, *trnF*-*GAA*_*ndhJ*, *ndhK*_*ndhC*, *accD*_*psaI*, *petA*_*psbJ*, and *ndhF*_*trnL*-*UAG* (Fig. S1). Most of the DNA sequence variation in *Engelhardia* species occurred in non-coding regions such as gene spacer region and gene intron region, and the sequence differentiation between LSC and SSC region was significantly higher than that in IR region (Figs. S1-2).

**Fig. 2:**
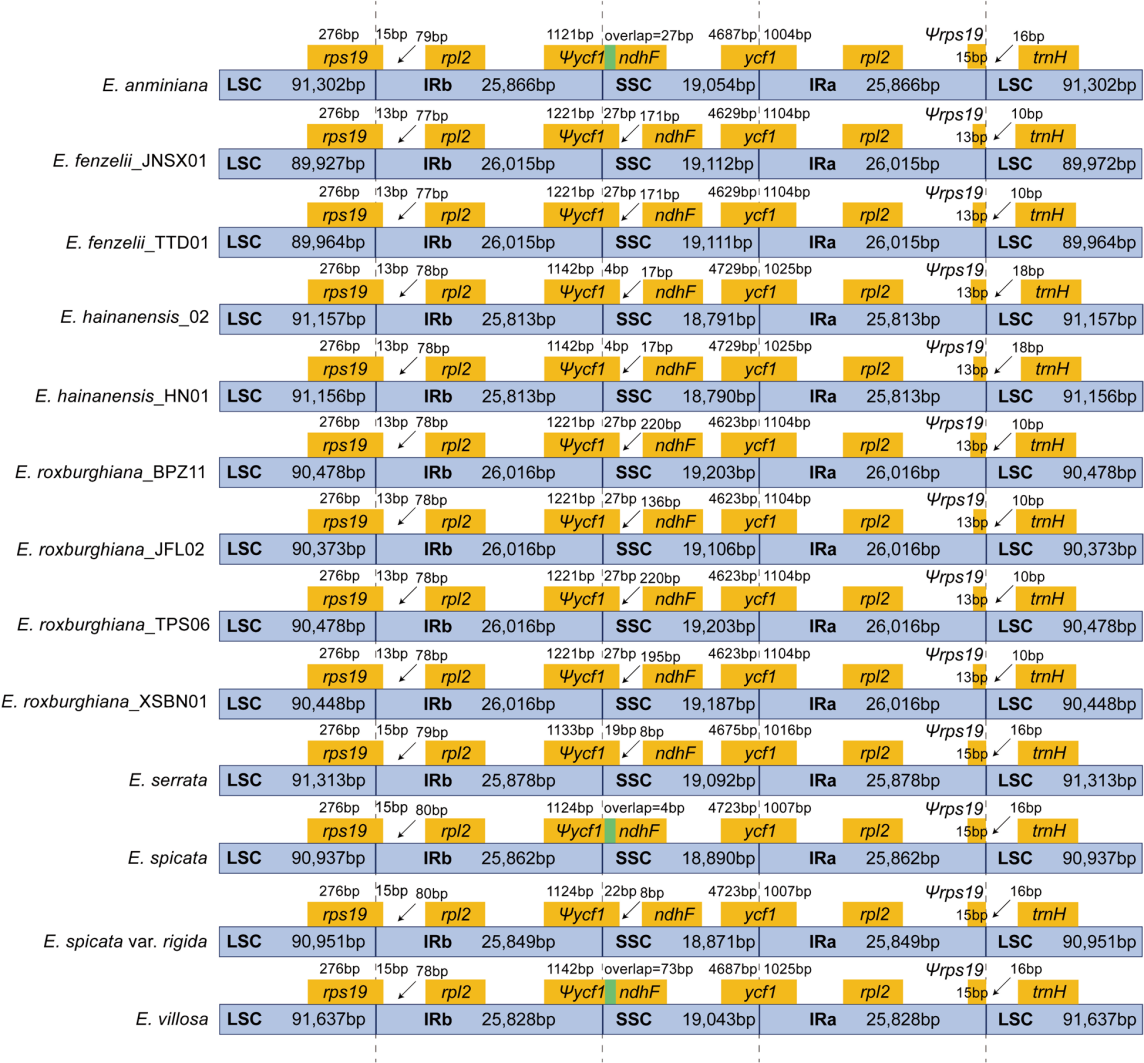
Comparison of the border positions of SSC, LSC, and IR regions among 13 *Engelhardia* plastomes. Genes close to or spanned the boundaries were shown in yellow boxes

By analyzing the boundary differences of LSC, SSC, IRa and IRb sequences in plastomes of *Engelhardia*, it was found the inner boundary differences were small. There was no large regional expansion and shortening of spacer regions occurred, which was consistent with the conserved character of plastomes within this genus (Fig. 2). The *ycf1* gene in all species spanned the SSC/IRa region, with the length of *ycf1* in SSC is 4623 bp–4729 bp, and that in IRa is 1004 bp–1104 bp. The pseudogene (*Ψycf1*) was formed at the corresponding position near the IRb/SSC boundary, and the extension of the short *Ψycf1* fragment into the SSC region was observed in all *Engelhardia* species. The overlapping of *Ψycf1* and *ndhF* has only been detected in *E. anminiana*, *E. spicata* and *E. villosa*. The *rps19* gene spanned the LSC/IRb regions in all the *Engelhardia* species, and it formed a pseudogene (*Ψrps19*) at the IRa/LSC boundary (Fig. 2).

### Repetitive sequences in *Engelhardia* plastomes

**Fig. 3:**
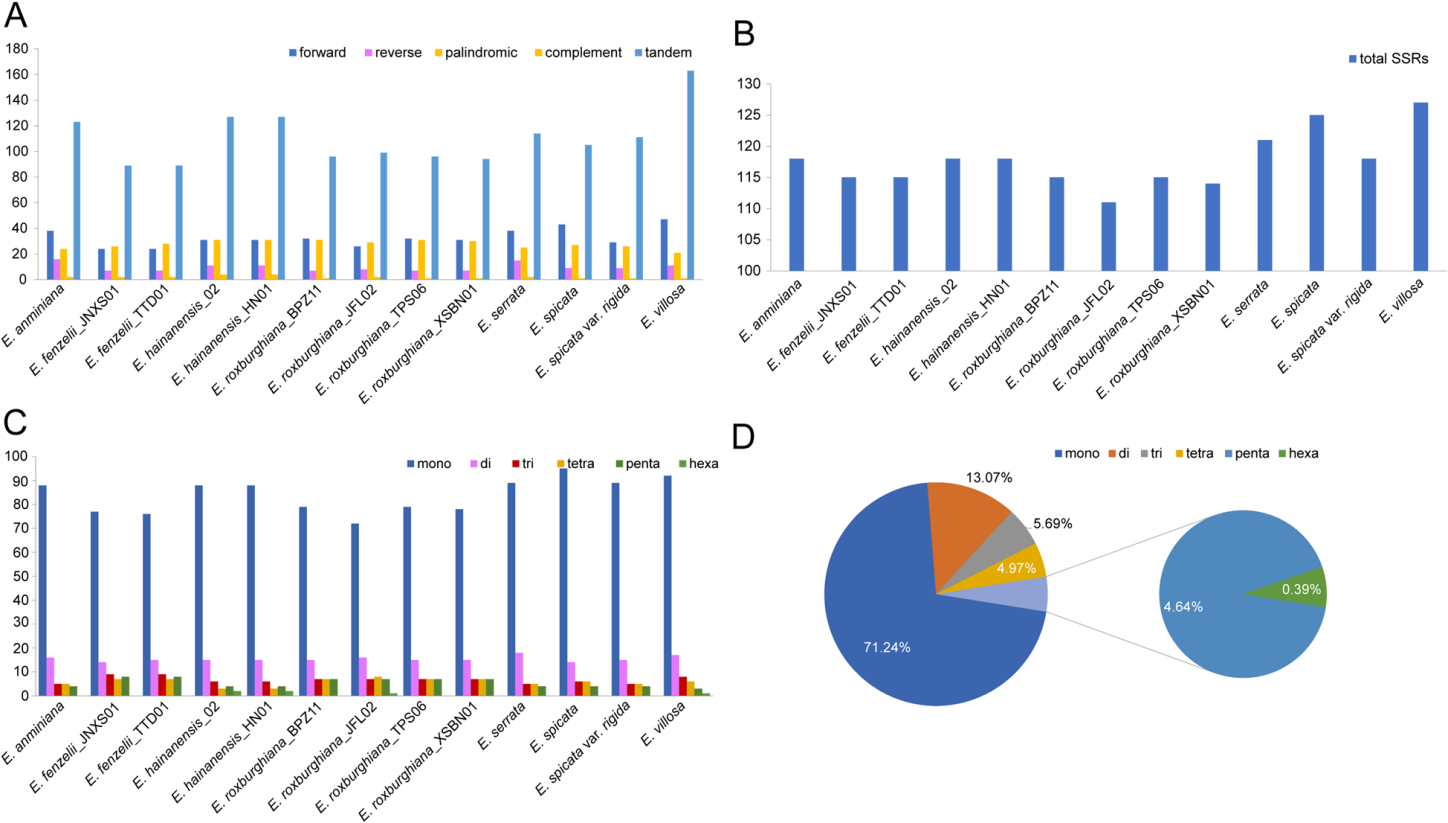
Analysis of repeated sequences in 13 *Engelhardia* plastomes. **A** Statics of dispersed repeat sequences. **B** Statics of simple sequence repeats (SSR). **C **Statics of different types of SSR. **D** Statics of the overall proportion of different types of SSR

Plastid genome repeats include dispersed repeats and tandem repeats. Dispersed repeats are further divided into four types: forward, reverse, complement and palindromic repeats. REPuter software identified 2,368 repeated sequences, including 24–47 forward repeats, 7–16 reverse repeats, 21–31 palindromic repeats, 1–4 complement repeats, and 89–163 tandem repeats, in the 13 *Engelhardia* plastomes (Table S4, Fig. 3). Most of the tandem repeats existed in non-coding regions such as IGS and introns (Table S4, Fig. S3). Overall, tandem repeats were more prevalent in *Engelhardia*, accounting for about 60.52% of all repeat types. On the contrary, the complement repeats were relatively small, accounting for 1.01% (Table S4, Fig. S4).

**Fig. 4:**
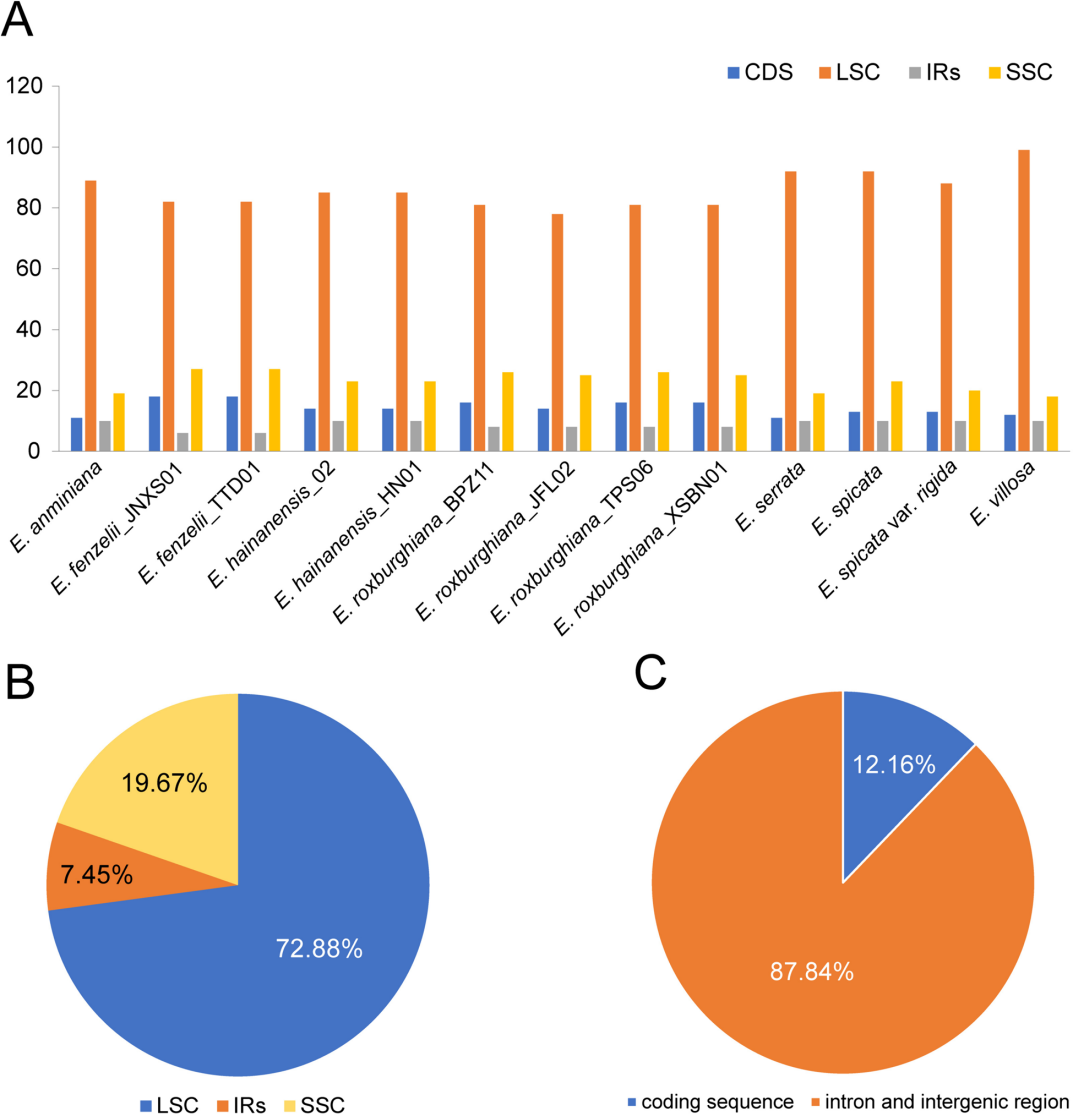
The distribution of simple sequence repeats (SSR) in 13 *Engelhardia* plastomes. **A** Statics of number of SSRs in the LSC, SSC, IR regions and in all the CDS. **B** Statics of the overall proportion of SSRs detected in different regions. **C** Statics of the overall proportion of SSRs detected in CDS and non-coding sequences

In this study, statistical analysis of SSR was performed by MISA online software, and a total of 1530 SSR loci were detected in the 13 *Engelhardia* plastomes. The total number of SSRs varied little among individuals, ranging from 111 (*E. roxburghiana*_JFL02) to 127 (*E. villosa*). The majority of these plastid SSR (ptSSR) were mono-nucleotide repeats, accounting for 71.24% of all SSR, followed by di- (13.07%), tri- (5.69%), tetra- (4.97%) and trinucleotide repeats (4.64%), while hexanucleotide repeats was the least, accounting for only 0.39% (Table S4, Fig. 3). The A/T type mononucleotide was the most abundant SSR, accounting for 98.44%, and only 17 G/C single nucleotide repeats were detected, which also resulted in the enrichment of A and T in the plastomes. Most SSRs were located in the LSC region (72.88%), and a smaller percentage of SSRs were distributed in the SSC (19.67%) and IR (7.45%) regions, respectively. Furthermore, most of the SSRs (87.84%) were distributed in the IGS and introns, while only 12.16% in the coding sequences (Table S4, Fig. 4).

### Comparative analysis of genomic variation in the *Carya*, *Engelhardia*, and *Juglans* plastomes

**Fig. 5:**
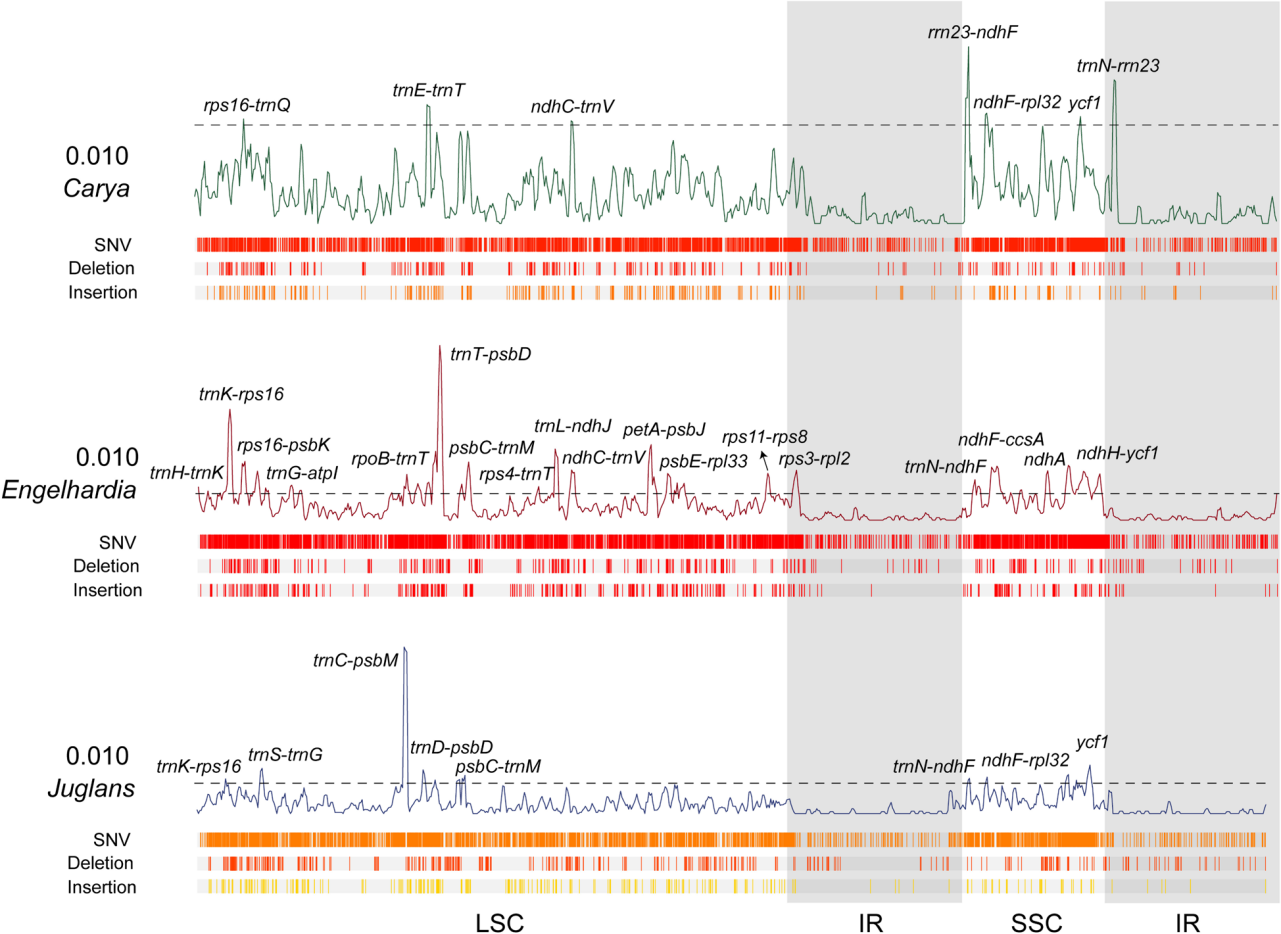
Nucleotide diversity and variation distribution of plastomes of *Carya*, *Engelhardia* and *Juglans*. The curved line depicts the fluctuation of π values across the genome alignment (dotted line marked the π values at 0.010), while the boxes below the curve represent the distribution of SNVs (top), Deletions and Insertions (bottom). The shadow layers in grey indicate the approximate range of IRs regions

Comparative analysis of nucleotide polymorphisms of *Carya*, *Engelhardia*, and *Juglans* plastomes, the variability in *Engelhardia* was higher than that of *Carya* and *Juglans* (Fig. 5). There were eighteen hypervariable regions in *Engelhardia* with Pi > 0.010 were *trnH-trnK*, *trnK*-*rps16*, *rps16*-*psbK*, *trnG*-*atpI*, *rpoB*-*trnT*, *trnT*-*psbD*, *psbC*-*trnM*, *rps4*-*trnT*, *trnL*-*ndhJ*, *ndhC*-*trnV*, *petA*-*psbJ*, *psbE*-*rpl33*, *rps11*-*rps8*, *rps3*-*rpl2*, *trnN*-*ndhF*, *ndhF*-*ccsA*, *ndhA* and *ndhH*-*ycf1*, while there were only seven and eight hypervariable regions in *Carya* and *Juglans*, respectively. Among them, *trnK*-*rps16*, *ndhF*-*rpl32*, and *ycf1* were common high variation hotspots in these three genera (Fig. 5).

Using *R. chiliantha* as a reference, we characterized genomic variations including single nucleotide variants (SNVs), insertions and deletions (InDels) in the plastomes of Juglandoideae and Engelhardioideae and found that they were very different among different species (Table S5a). A total of 115,213 SNVs, 9502 insertions (1–274 bp) and 10,428 deletions (1–2,468 bp) were identified in all the collected species (Table S5d). The number of SNVs, deletions and insertions per kb varied at the plastid genome level, with the average values of 15.03, 1.36 and 1.24 in Juglandaceae, 11.84, 1.04 and 0.93 in *Carya*, 17.48, 1.48 and 1.64 in *Engelhardia*, and 17.20, 1.70 and 1.28 in *Juglans*, respectively. In terms of these three types of genomic variation, the IR regions presented the fewest numbers per kb, with the average values of 1.71, 0.15 and 0.09 in Juglandaceae, 1.81, 0.13 and 0.11 in *Carya*, 1.66, 0.20 and 0.10 in *Engelhardia*, and 1.70, 0.14 and 0.05 in *Juglans*, respectively. The LSC regions exhibited the maximum numbers of SNVs, deletions and insertions per kb with the average values of 9.04, 0.97 and 0.93 in Juglandaceae, 6.17, 0.70 and 0.63 in *Carya*, 10.62, 1.01 and 1.23 in *Engelhardia* being, and 11.30, 1.30 and 1.04 in *Juglans* (Table S5b). These results collectively indicate that the IR regions were more conserved than the single-copy regions.

All genomic structural variations were mapped onto a phylogenetic tree constructed based on the plastid genome, with very different times of insertion events and deletion events occurring in *Carya* (insertion events: 132–199 times; deletion events: 135–236 times), *Engelhardia* (186–364; 155–311), and *Juglans* (149–230; 192–331). So,the structural variation of *Carya* was less than that of *Engelhardia* and *Juglans*. The range of structural variation among *Engelhardia* species was relatively large, especially in *E. serrata* and *E. villosa*, with 329 insertions and 306 deletions in *E. serrata*, and 364 insertions and 311 deletions in *E. villosa* (Fig. S5).

**Fig. 6:**
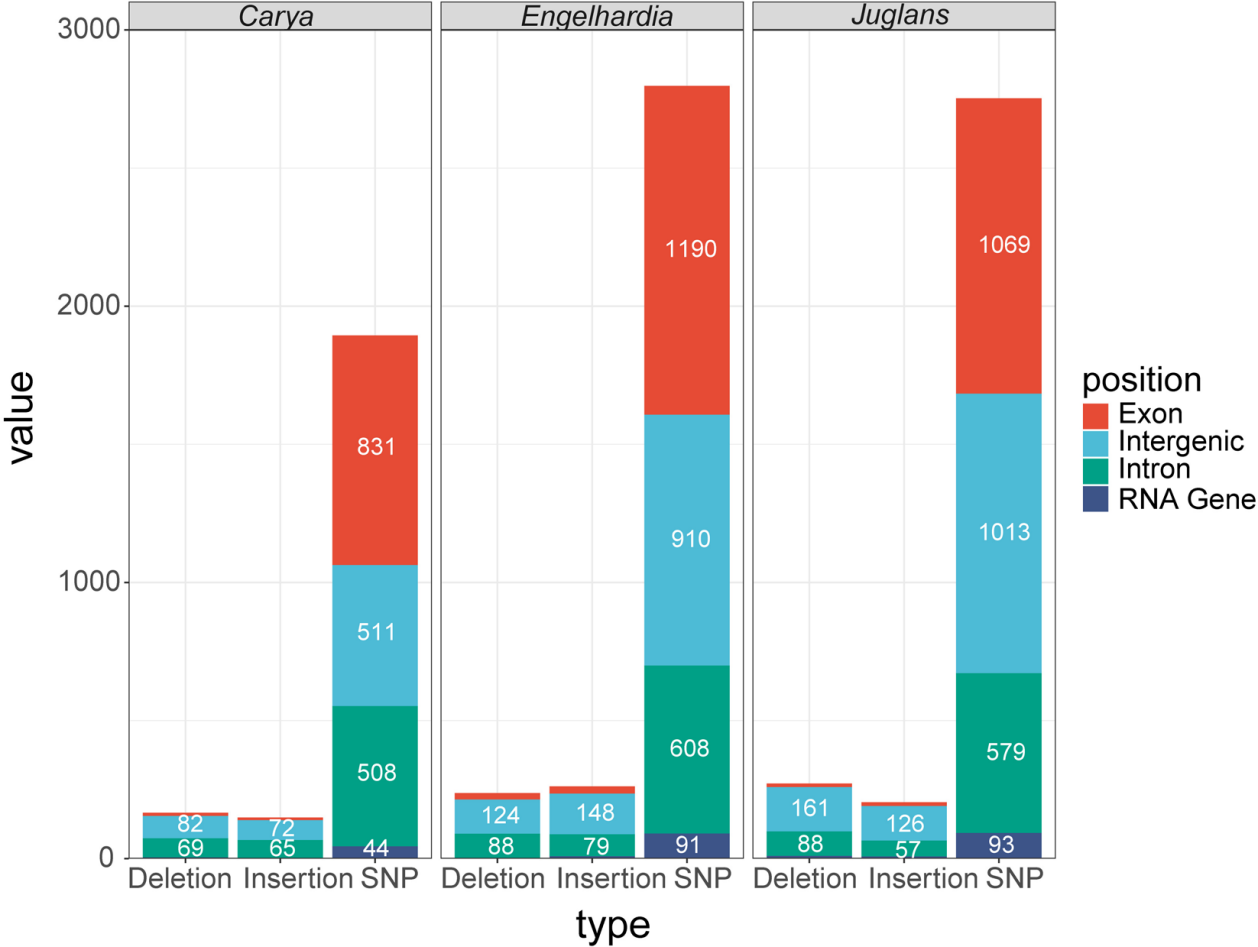
The average number of SNVs, Deletions and Insertions located on the gene intervals, exons, introns, and RNA genes of plastomes of *Carya*, *Engelhardia* and *Juglans*

The corresponding genomic positions of these identified InDels were mapped and located into these plastomes of Juglandoideae and Engelhardioideae. It was found that 90% of InDels were found in intronic (35%) and intergenic regions (55%) for Juglandaceae, 92% of InDels were found in intronic (43%) and intergenic regions (49%) for *Carya*, 88% of InDels were found in intronic (33%) and intergenic regions (55%) for *Engelhardia*, and 91% of InDels were found in intronic (31%) and intergenic regions 60%) for *Juglans* (Table S5c; Fig. 6).

### Codon usage analysis of Juglandaceae plastomes

Comparing the coding sequences of 50 genes with a length over 300 bp of Juglandaceae plastomes, it was found that there were two codons with RSCU value of 1, which were AUG and UGG encoding methionine (Met) and tryptophan (Trp)(Table S6a). There were 29 codons with RSCU > 1, of which 16 end with U and 12 end with A, which was the same in Engelhardioideae, Juglandoideae and Rhoipteleoideae (Table S6a). The codons ending with U or A were the preferred codons in the plastomes of these three subfamilies (Fig. S6). There was no significant difference in the codon bias of the genes of most Juglandaceae plastomes (Fig. S6). However, the A/T content of the third base in the coding sequence was significantly higher than the G/C content, T3s (0.4748–0.4782) > A3s (0.4399–0.4438) > G3s (0.1695–0.1722) > C3s (0.1613–0.1649) (Table S6b). We found that *Carya ovata* and *Carya palmeri* had the highest values of ENC, GC3s and GC, while *Platycarya strobilacea* had the lowest values. No significant differences in codon preferences within genera were detected among these three subfamilies (Table S6b).

The codon usage pattern parameters, ENC, Fop, CBI and CAI of the coding genes of three subfamilies were further calculated and plotted (Table S6c). The CAI values were between 0.09 and 0.31, *psbA*, *rbcL* and *psbD* with the highest CAI value, and *rpl20*, *rpl18* and *rps8* with the lowest one. Most of the CBI values ranged from -0.23 to 0.23, the highest were *psbA*, *psbD* and *rbcL*, and the lowest were *ndhF*, *ndhG* and *rps14*. Most of the Fop values were between 0.26–0.55, the highest were *psbA*, *psbD* and *rbcL*, and the lowest were *ndhG*, *ndhF* and *petD*. Most of the ENC value were concentrated between 35.71 and 60.6, the highest were *ycf3*, *ycf2* and *rpl2,* and the lowest were *rps18*, *petD* and *rps14* (Table S6c).The highly expressed genes in the plastid genome of three subfamilies were *ycf2*, *rpoC1* and *rpoC2*, and the low expressed genes are *rps18*, *petD* and *rps14* (Table S6d). Combined with the 29 high-frequency codons with RSCU value > 1 in Table S4a, 10 common optimal codons were finally determined, which were CUU, GUU, UCU, UCA, CCU, CCA, GCU, AAU, CGA, GGA, and all end with A or U (Table S6d).

There was a positive correlation between the codon preference index (CBI) and the optimal codon usage frequency (Fop), and the highest correlation coefficient of 0.97 (Table S6e). The correlation coefficients between CAI and CBI and between CAI and Fop were also higher, which were 0.72 and 0.76, respectively, showing a positive correlation. In addition, there were negative correlation between T3s/C3s, T3s/A3s, T3s/G3s, T3s/GC3s, T3s/GC, C3s/A3s, C3s/G3s, A3s/G3s, A3s/CAI, A3s/CBI, A3s/Fop, A3s/ENC, A3s/GC3s, A3s/GC, G3s/CAI, G3s/CBI, G3s/Fop, CAI/GC, etc. Among them, A3s/CAI showed the highest degree of negative correlation, with a correlation coefficient of -0.57 (Fig. S7). The results of three subfamilies were similar to the whole Juglandaceae family, with the highest correlation coefficients being CBI and Fop, followed by the correlation coefficients between CAI and CBI, as well as between CAI and Fop (Fig. S7). The ENC values were positively correlated with T3s, C3s, G3s and GC3s. However, ENC values were negatively correlated with A3s. Our results showed that the content of the third base of synonymous codons was closely related to gene expression levels, and T3s, C3s and G3s were positively correlated with gene expression, while A3s was negatively correlated with gene expression (Table S6e, Fig. S7).

**Fig. 7:**
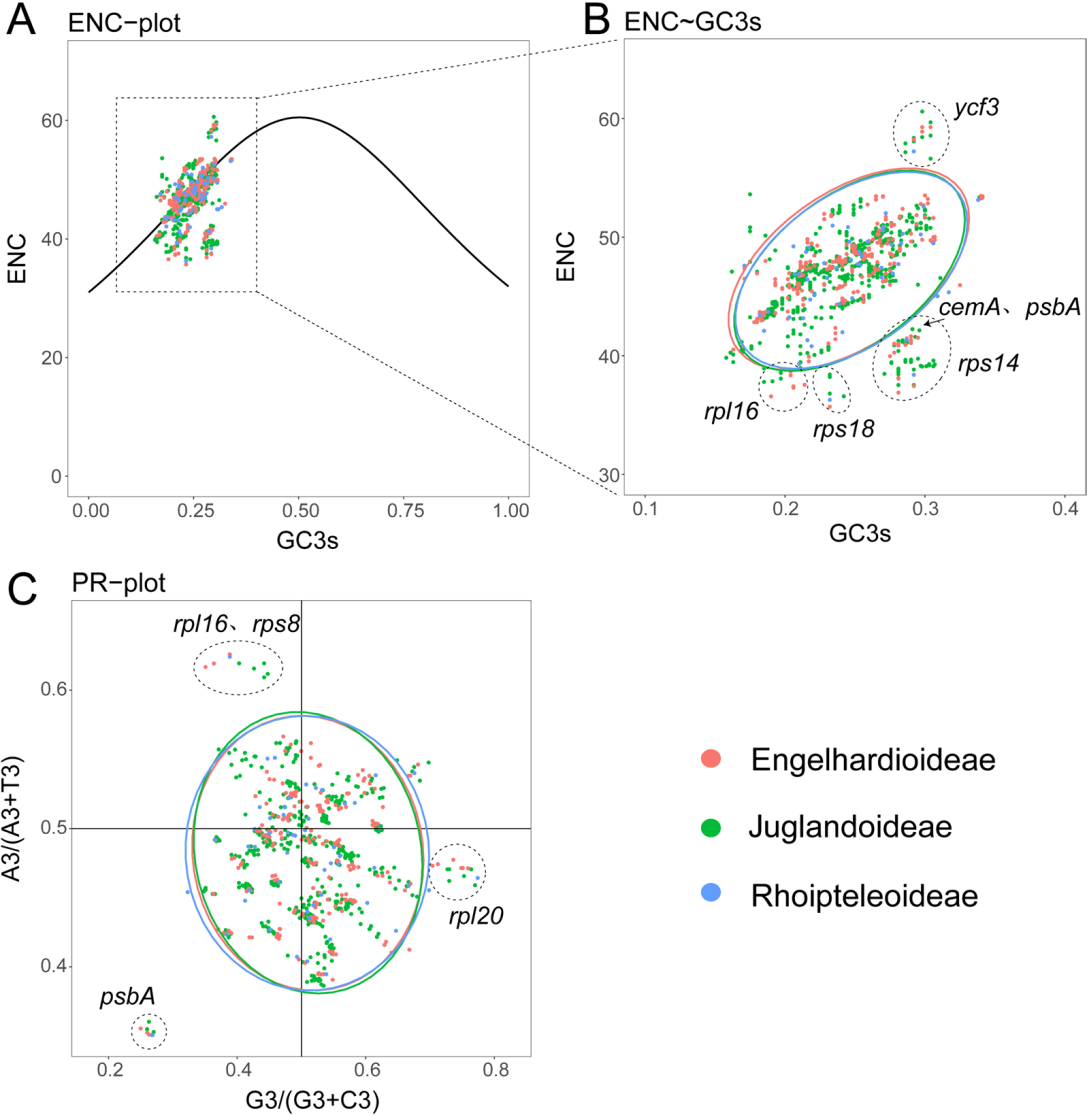
ENC and PR2-plots of protein-coding genes in plastomes of 50 Juglandaceae species. **A** ENC plots showing observed and expected and ENC values vs. GC3s values of protein-coding genes in these plastomes. **B** Comparison of ENC differences in two different climate zones. **C** PR2-plots showing the base composition characteristics of protein-coding genes in the 50 Juglandaceae plastomes. Red, genes of species from the Engelhardioideae species; Green, genes of species from the Juglandoideae species; Blue, genes of species from the Rhoipteleoideae species

ENC values for all screened gene coding sequences ranged from 35.71 to 60.6. The ENC frequencies were calculated using the formula (ENCexp-ENCobs)/ENCexp, ranging from -0.25 to 0.28. There were 2051 ENC frequencies in the range of -0.1 to 0.1, which were close to the expected ENC values (Table S6f). Based on the standard curve formula ENC = 2 + GC_3_ + 29/[GC_3_^2^ + (1 − GC_3_)^2^], we took ENC as the ordinate and GC3s as the abscissa to draw a scatter plot (Fig. 7). It was found that most of the genes were located on or near the standard curve (Fig. 7A). However, we also found the observed ENC values of six genes (*rpl16*, *rps18*, *cemA*, *psbA*, *rps14*, and *ycf3*) in all species deviated significantly from the standard curve (Fig. 7A, B). Among all genes, *ycf3* showed the highest ENC value, while *rps*18 and *rpl*16 showed the lowest ENC value (Fig. 7B; Table S6f).

PR2-plot is used to analyze the composition of the four bases at the third position of the codon encoding the amino acid, plotting with G3/(G3 + C3) and A3/(A3 + T3) as the horizontal and vertical coordinates. The results showed that A/T and G/C (pyrimidine versus purine) were used slightly differently at the third codon position in the protein-coding sequence of Juglandaceae (Fig. 7C). The PR2-plot showed that there was a slight imbalance in the use of A/T and G/C at the third codon of the CDSs of 36 Juglandaceae plastomes, especially the four CDSs (*psbA*, *rpl20*, *rpl16* and *rps8*) (Fig. 7C).The number of genes in the third and fourth quadrants was more than that in the first and second quadrants, and the number of genes distributed in the fourth quadrant was greater than the number of genes distributed in the other three quadrants, so G and T were used most frequently (Fig. 7C).

### Selective pressure analysis of CDS in Juglandaceae

**Fig. 8:**
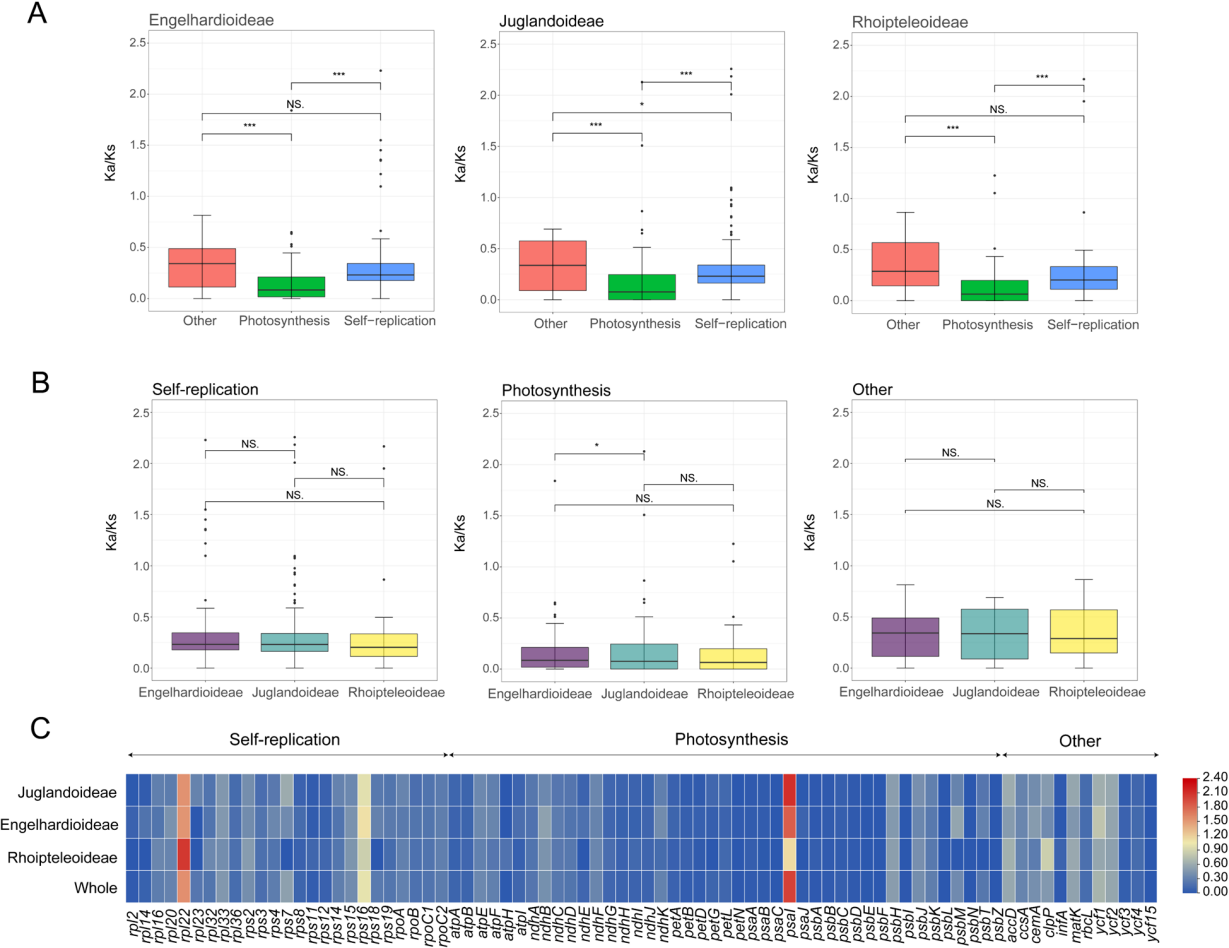
Analyses of evolutionary pressure on plastid gene homologues in 50 Juglandaceae species. **A** A comparison of *Ka/Ks* values among photosynthesis-correlated genes, self-replication-correlated genes, and other protein coding genes in the three subfamilies. **B** A comparison of *Ka/Ks* values among gene homologues from the three subfamilies for photosynthesis-correlated genes, self-replication-correlated genes, and other protein coding genes. *, *p* < 0.05; **, *p* < 0.01; ***, *p* < 0.001,; NS, *p* > 0.05. **C** A heatmap showing the Ka/Ks values of CDS genes within the Juglandoideae, Engelhardioideae and Rhoipteleoideae subfamily

To analyze the evolutionary pressure among the protein coding sequences of eight Juglandaceae species, the *Ka/Ks* value of 80 protein coding sequences (CDS) were calculated. The results showed that the *Ka/Ks* values of 78 genes was almost all less than 1, with only *rpl22* and *psaI* showing *Ka/Ks* > 1 We also found that *rps16* was only subjected to positive selection in Juglandaceae and Engelhardioideae. For all Juglandaceae samples, the *Ka/Ks* values of photosynthesis-related genes were significantly lower than those of self-replication-related and other genes (Fig. 8A, Table S7b). For functional classification genes, except for differences in photosynthesis related genes between Engelhardioideae and Juglandoideae, other Ka/Ks values showed no significant differences (Fig. 8C, Table S7c).

### Phylogenetic analysis of Juglandaceae

In this study, *Quercus rubra* (Fagaceae) was used as the outgroup, ML and BI trees of Juglandaceae species based on the complete plastomes (excluding one copy of the inverted repeat) showed nearly identical topologies (Fig. S8). The phylogenomic results indicated that Juglandaceae family was mainly divided into three groups, including Juglandoideae, Engelhardioideae and Rhoipteleoideae subfamilies, with a very high support rate (BS = 100%, PP = 1). The phylogenomic tree further supported 7 major branches, corresponding to 7 genera, namely the monophyletic *Carya*, *Juglans*, *Pterocarya*, *Cyclocarya*, *Platycarya*, *Engelhardia* and *Rhoiptelea*.

There were two main clades in Juglandoideae, clade I was *Carya*, clade II were *Juglans*, *Pterocarya*, *Cyclocarya* and *Platycarya*. In the ML tree, the support rate inside clade I (BS = 63–100%) was lower than that of clade II (BS = 66–100%). The species of *Carya* were divided into two sects, *C. hunanensis*, *C. kweichowensis*, *C. sinensis*, *C. polianei*, *C. tonkinensis*, and *C. cathayensis* were grouped together, while the remaining 12 species were grouped together. The *Juglans* were divided into three sects, namely the Sect. *Juglans* or *Dioscaryon*, Sect. *Cardiocaryon*, and the Sect. *Rhysocaryon*. The Sect. *Juglans*/*Dioscaryon* included *J. regia* and *J. sigillata*, while the Sect. *Cardiocaryon* included *J. mandshurica*, *J. ailanthifolia* and *J. hopeiensis*, the Sect. *Rhysocaryon* included *J. cinerea*, *J. nigra*, *J. hindsii*, *J. major* and *J. microcarpa*. The *Pterocarya* were divided into two sects, one included *P. fraxinifolia*, *P. stenoptera* and *P. hupehensis*, the other included *P. macroptera* var. *insignis* and *P. tonkinensis*. *Cyclocarya paliurus* was a single species of *Cyclocarya*, which was closely related to *Pterocarya* according to the phylogenetic relationships.

The species of the Engelhardioideae were closely related and were further divided into two main clades, which was consistent with the Sect. *Engelhardia* (Clade I) and Sect. *Psilocarpeae* (Clade II), with a very high support rate (BS = 100%, PP = 1). The Clade I included *E. spicata*, *E. spicata* var. *rigida*, *E. hainanensis*, *E. serrata*, *E. anminiana*, and *E. villosa*. The clade II included *E. roxburghiana* and *E. fenzelii*, which were sister species. The Rhoipteleoideae subfamily only included *R. chiliantha*, a single genus and single species.

### The divergence time and historical diversification of Juglandaceae

**Fig. 9:**
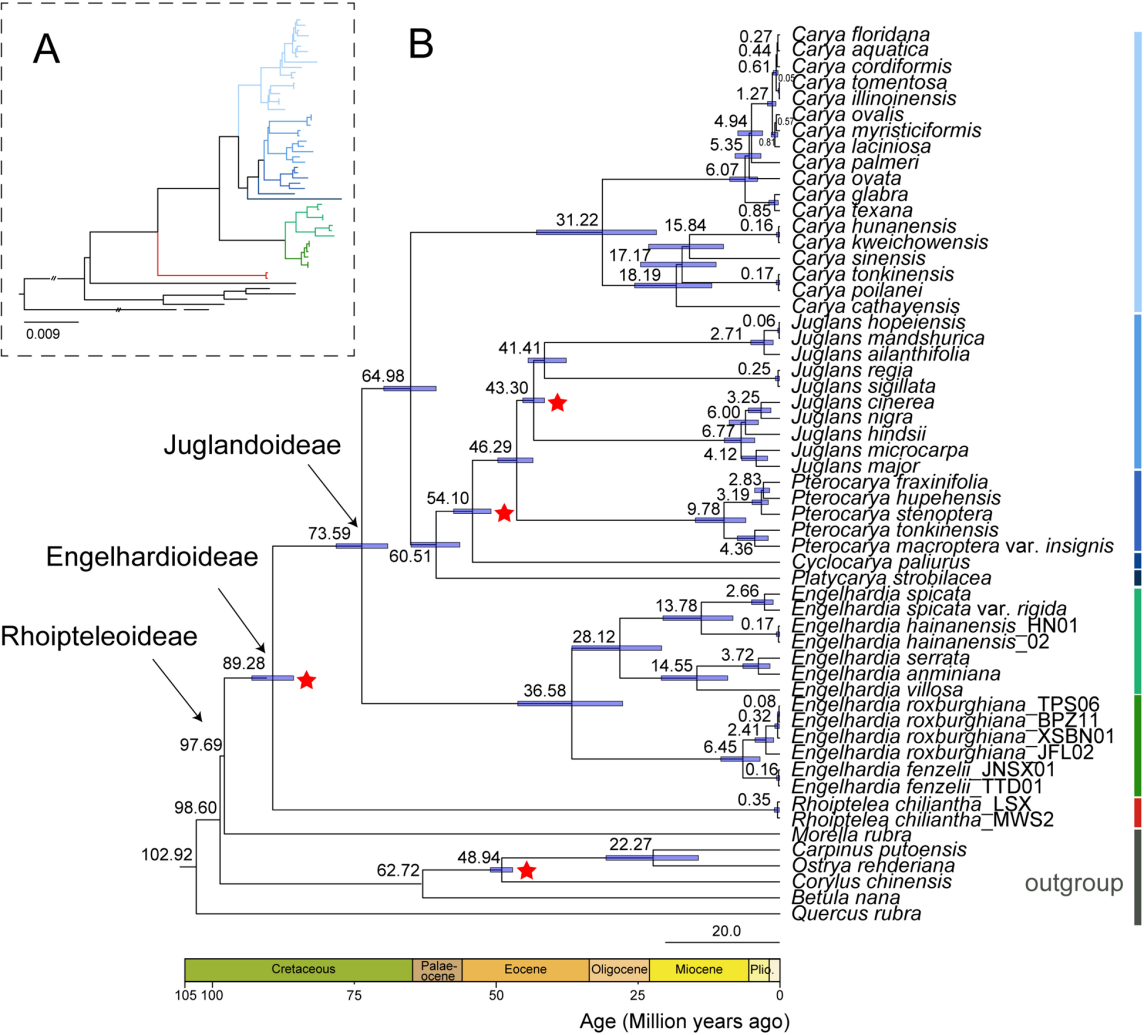
The time-calibrated phylogenetic tree of Juglandaceae based on 80 protein-coding genes of plastomes. Estimated mean divergence times using a relaxed molecular clock model with 4 fossil priors (red stars). The blue bar on the node represents the estimated 95% HPD intervals around the mean divergence time. Nodes are numbered by ages. The genera and subfamilies of Juglandaceae are shown in the figure

By using multiple fossil correction points to estimate the differentiation time of the Juglandaceae family, the results showed that the crown nodes of Juglandaceae were approximately 97.69 Mya (95% highest posterior density (HPD): 95.49 Mya–100.58 Mya), which differentiated from Myricaceae in the Early Cretaceous period (Fig. 9). The three subfamilies, namely Rhoipteleoideae, Engelhardioideae and Juglandoideae, successively diverged at 89.28 Mya (95% HPD: 85.6–92.96 Mya; Middle Cretaceous) and 73.59 Mya (95% HPD: 69.01–78.13 Mya; Late Cretaceous) (Fig. 9).

Most genera of Juglandaceae were diverged between 46.20 and 73.59 Mya. The differentiation time of the two clades in the Engelhardoideae subfamily was approximately between 27.64 and 46.11 Mya, mainly occurring from the Early Eocene to the Middle Oligocene. The phylogeny and divergence times didn’t support rapid radiation occurred in the evolution history of *Engelhardia*. In the Juglandoideae subfamily, the crown age of *Carya* was estimated at 64.98 Mya (95% HPD: 60.49–69.70 Mya), *Platycarya* at 60.51 Mya (95% HPD: 56.32–64.91 Mya) during the Late Paleocene, and *Cyclocarya paliurus* at 54.10 Mya (95% HPD: 50.84–57.41 Mya). The divergence of *Pterocarya* and *Juglans* was estimated at 46.29 Mya (95% HPD: 43.43–49.63 Mya) during the Middle Eocene. Most genera of Juglandoideae subfamily were diverged between 46.29 and 64.98 Mya in the relatively warm and dry climate of the Middle Paleocene to the Early Eocene (Fig. 9).

## Discussion

### Comparative analysis of *Engelhardia* plastomes

In this study, the plastomes of 13 individuals from eight species *Engelhardia* were newly sequenced, annotated and compared. The results showed that all species of the *Engelhardia* had a typical tetrad structure, and the genome sizes were similar, about 161 kb (161,069 bp–162,336 bp), and the GC contents of the plastomes were 35.8%–36.0%, which were similar to the sequence length and composition of the previously obtained Juglandaceae plastomes [1, 25–27]. By comparison of the GC content of each part of the plastomes of *Engelhardia*, it was found that the GC content in the IR regions was higher than those of the LSC and SSC regions, and the high GC content is conducive to the stability of the genome, so the conservation of the IR regions may be related to the GC content [28]. Multiple plastomes comparison among all the *Engelhardia* species using mVISTA and Mauve alignment showed a very good synteny. No inversion or translocation of DNA fragment rearrangement was detected in the sequences, which is consistent with the conservation of the plastomes [29, 30]. By analyzing the boundary differences of LSC, SSC, IRa, and IRb sequences in the plastid genome of *Engelhardia*, it was found that the boundary differences of plastomes of *Engelhardia* were small (Fig. 2), which is similar to *Carya*, with a relatively-conserved pattern of IR boundaries [26]. In *Engelhardia*, the *ycf1* gene contains two copies, one located at the SSC/IRa boundary is a complete gene, the other located at the IRb/SSC boundary in the form of a pseudogene *Ψycf1*, which no longer encodes a protein [31]. Likewise, due to the existence of the boundary effect, *Ψrps19* existed at the IRa/LSC boundary (Fig. 2).

Repeated sequences are ubiquitous in gene sequences and gene intervals, which not only protect the coding sequence [32], but also maintain the stability of the genome. The SSRs of the plastid genome have abundant polymorphic sites and are widely used in molecular markers, variety identification and other studies [33, 34]. The present study examined dispersed repeats and tandem repeats, found that tandem repeats were more common (60.52%) in *Engelhardia*. In our study, 24 complement repeats were detected in *Engelhardia*, this result was consistent with other Juglandaceae species [35–37]. Almost all repeating units of SSRs consist of A and T bases, which is consistent with the previous study by Yi et al*.* [38]. These plastomes SSRs can provide candidate molecular markers for *Engelhardia*, which will contribute to population genetics and evolution studies, as well as its molecular breeding and conservation.

DNA barcoding is a novel species identification technology that uses standard short gene regions as markers for rapid, accurate and efficient identification of species [39]. Zhang et al*.* used five plastome regions (*psbA-trnH*, *trnL-trnF*, *rps16*, *trnS-trnG*, and *rpl32-trnL*), one nuclear DNA region (nrITS), and 11 nuclear simple sequence repeats (nSSR) were selected for species identification of *Engelhardia* [9]. In our study, we used the complete plastomes to carry out nucleotide polymorphisms analysis of *Engelhardia* for more potential molecular markers. The results showed that the IR regions of all species had lower genetic polymorphism than the LSC and SSC regions, and the coding region sequences were more conserved than the non-coding region sequences (Fig. 5), which were similar to the findings of most angiosperms [40]. However, we still found eighteen hypervariable regions in *Engelhardia*, including *trnK*-*rps16*, *ndhF*-*rpl32*, and *ycf1*, which also were highly variable in *Carya* and *Juglans* (Fig. 5). They could be used for species identification of *Engelhardia*, even for Juglandaceae.

### Genomic structural variation across plastomes of two subfamilies

With *R. chiliantha* as reference, we characterized genomic variations including SNVs, insertions and deletions (InDels) in the plastid genomes of Juglandoideae and Engelhardioideae. Although they were different among different species (Table S5a), the overall was conserved. By comparing genomic variations in different regions, we found that the IR region has the smallest number of variations per kb (Table S5a), was more conserved than the single copy regions, and genes were more conserved than the intergenic regions, which is consistent with the characteristics of the genome [40]. Localizing these mutations into plastome, it was found that the majority of insertions and deletions were distributed in introns and intergenic regions. The uneven distribution of genomic structural variations in plastids suggested that they may have negative effects and can be easily eliminated through purification selection [41].

Structural variation may not only affect the heterogeneity of genomic structure, but also affect the evolution of protein coding genes in the plastomes of Juglandoideae and Engelhardioideae. By length analysis of InDels in protein coding gene, it was found that only 1200 were multiples of 3 in length among the 3993 InDels in *Engelhardia*, while there were 1428/5670 and 1248/4764 InDels for *Carya* and *Juglans*, respectively. This finding showed that negative selection of frameshift InDels may not truly affect the plastid protein coding genes, which is contrary to the results observed in the six nuclear genomes of other flowering plants [42]. By mapping structural variations to the exons and RNA genes of protein coding genes in the plastomes, the universality of plastid genome structural variations has been further confirmed, suggesting that InDels may be an important driving factor for the evolution of plastid genes in Juglandoideae and Engelhardioideae.

### Codon usage bias and gene evolution in Juglandaceae plastomes

The codon usage pattern of the Juglandaceae plastomes plays an important role in exploring its evolutionary process [43]. In our study, codon bias of Juglandaceae plastomes were investigated. The frequency of usage of multiple synonymous codons encoding the same amino acid is not equal, a phenomenon known as codon usage bias [44]. The relative synonymous codon usage (RSCU) can directly reflect the preference of codon usage [45]. So RSCU values of all selected plastomes were calculated. We found that the third base of most codons ends with A or U (Table S6a), this result is consistent with the results of studies such as *Crataegus* [46], *Pisum* [47], and *Miscanthus* [48], indicating that the third base in the plant plastomes may have similar usage patterns [49]. By constructing high and low expressed gene sets, codons with RSCU > 1 and ΔRSCU > 0.08 were defined as optimal codons. Then, nine optimal codons of Juglandaceae plastomes were determined, all ending with A or U (Table S6d). In general, G and C (or A and T) are distributed proportionally at the third codon base, indicating that the codon usage bias of the species is affected by mutational pressure [50]; if disproportionately distributed over the third base of a codon, it indicates that the codon usage bias is influenced by natural selection pressure [51]. Therefore, it was speculated that the codon bias in the plastid genome sequence of Juglandaceae was not only affected by base mutations, but also affected by natural selection pressure. Genes from Engelhardioideae, Juglandoideae and Rhoipteleoideae species were presented in different colors in ENC and PR2-plots (Fig. 7). There was no significant potential difference in the main driving forces of codon use bias among these three subfamily plants (Fig. 7; Table S6).

We found a positive correlation between the codon preference index (CBI) and the optimal codon usage frequency (Fop), with the highest correlation coefficient of 0.97, indicating that the codon usage pattern in the plastomes of Juglandaceae may be determined by the optimal codon usage frequency during evolution [43]. In the Juglandaceae and two subfamilies (Juglandoideae, Engelhardioideae), the ENC values were positively correlated with T3s, C3s, G3s and GC3s. However, ENC values were negatively correlated with A3s. ENC values can be used to determine the relative expression levels of genes [52], so we inferred that the content of the third base of synonymous codons in Juglandaceae and two subfamilies (Juglandoideae, Engelhardioideae) was closely related to gene expression levels, and T3s, C3s and G3s are positively correlated with gene expression, while A3s was negatively correlated with gene expression (Table S6e, Fig. S5). In the Rhoipteleoideae subfamily, C3s, G3s, and GC3s were positively correlated with gene expression, while T3s and A3s were negatively correlated with gene expression (Fig. S6).

There were 50 protein-coding genes with lengths longer than 300 bp in the plastomes of Juglandaceae. The ENC values of these screened gene coding sequences ranged from 35.71 to 60.60. According to the ENC values ranging from 20 (completely biased) to 61 (unbiased) [53], and when the ENC value is less than 35, the codon usage of the gene or genome has a strong bias [54]. Based on these two characteristics, we found that the codon usage bias of protein-coding genes in Juglandaceae plastids was weak. There were 2051 ENC frequency ratios between -0.1 and 0.1 (Table S6f), which were close to the expected ENC value, indicating that the difference between the expected ENC value and the actual values of most genes are small. The results showed that the content of bases at the third position of synonymous codons was closely related to gene expression. The GC content of the third base of codons (GC3s) is considered to be most likely to directly reflect codon usage patterns [55] and may be an important factor leading to codon usage bias. The scatter plot was drawn with ENC as the ordinate and GC3s as the abscissa to explore the main features of codon usage (Fig. 7). When the scatter points are on or near the standard curve, it indicates that the codon preference is affected by mutational pressure, and vice versa, it indicates that the codon usage preference is affected by factors such as natural selection [56]. It was found that most of the scatter points were located on or near the curve (Fig. 7), indicating that the mutation had a greater effect on codon bias. Further ENC-plot analysis showed that the ENC values of most genes were close to the expected value (Fig. 7A), suggesting that the codon usage biases of these genes were related to GC3, and mutation was the main factor influencing factor. In addition, some genes (*rpl16*, *rps18*, and *rps14*) were much lower than the expected curve (Fig. 7), which also confirmed the influence of natural selection on codon preferences of these genes.

Due to the influence of natural selection and base mutation, PR-plot drawing analysis can show the preference of coding genes in the genome in the use of the third codon base. If the base mutation occurs in the third codon, the proportion of synonymous codons AT and CG in the gene or genome is equal. Conversely, if there is selective pressure, some codons "preferred" for translation will be used more frequently [57]. The PR-plot analysis of the Juglandaceae and three subfamilies showed that the selection of A/T and G/C at the third base of the protein-coding sequence was different, and the frequency of using G and T (purine) bases was higher(Fig. 7C), indicating that it was mainly influenced by selection pressure. Based on ENC-plot analysis and PR-plot analysis, natural selection and mutation jointly affect the codon usage patterns of Juglandaceae plastomes, with mutational pressure playing a major role, which is consistent with the results of *Oncidium* Gower Ramsey [58].

Synonymous and non-synonymous nucleotide substitution patterns are valuable for gene evolution studies [59]. Due to the effect of purification selection, the substitution rate of non-synonymous nucleotides is lower than that of synonymous nucleotides, so the ratio of *Ka/Ks* is less than 1 in most cases [60]. In order to gain a clear understanding of the adaptive evolution of the Juglandaceae plastomes, we calculated the *Ka/Ks* ratios of protein-coding genes [41]. Our results showed that only the the Ka/Ks ratio of *ycf1* was greater than 1, and the Ka/Ks ratios of the remaining 79 gene was less than 1, indicating a strong purification selection pressure (Table S7a). We also noted that *rps16* was only in positive selection in Juglandaceae and Engelhardioideae. As a self-replication related gene in plant plastid organelles, *rps16* is essential for plant survival [61]. The positively selected *rps16* gene may play a key role in the process of adaptation of Engelhardioideae species. There were differences in photosynthesis-related genes between Engelhardioideae and Juglandoideae subfamily (Fig. 8C, Table S7b), which may be due to differences of photosynthetic adaptation between temperate subfamily Juglandoideae and tropical subfamily Engelhardioideae [2]. The distribution of these genes to the plastomes indicated that most genes contained in the SSC and LSC regions experienced greater selection pressure than other plastid genomic regions, while the IR regions were more conserved. In addition, genes with different functions evolve with different rates, and the selection pressure of genes involved in photosynthesis in plastome is often lower than that of genes related to self-replication and other functions, resulting in differences in gene expression and function [62] (Fig. 8).

### Phylogenetic relationships of Juglandaceae

Plant taxonomy are traditionally based on morphological characteristics, but the morphology is often affected by factors such as environment and parallel evolution [63], so molecular evidence is also needed. Based on nuclear genes and plastid gene fragments, predecessors have carried out related research on the phylogenetic relationship of *Engelhardia* [9, 10], but these plastid gene fragments do not have enough information to distinguish closely related species. In our study, ML and BI phylogenetic trees were constructed based on 50 accessions of Juglandaceae and 6 species from Myricaceae, Betulaceae and Fagaceae (Fig. S8). The phylogenetic tree constructed based on two different algorithms, ML and BI, presented almost identical topological structures.

The Juglandaceae was divided into three groups, including Juglandoideae, Engelhardioideae and Rhoipteleoideae subfamily [64], and had a very high support rate (BS = 100%, PP = 1) (Fig. S8). First, the five main branches of Juglandoideae subfamily correspond exactly to five genera, namely *Carya*, *Juglans*, *Pterocarya*, *Cyclocarya*, and *Platycarya*, all of which had high support rates (BS = 100%, PP = 1). According to the fruit morphology, these five genera were divided into two categories, including winged and wingless, namely *Pterocarya*, *Cyclocarya*, and *Platycarya* belong to winged types, while *Carya* and *Juglans* belong to wingless types [64]. According to the phylogenetic tree results, it was found that the phylogenetic relationship between the *Juglans* and *Pterocarya* was closer. Although the fruit morphology of the two genera was completely different, the fruit morphology of *Carya* and *Juglans* was similar, the phylogenetic relationship is distant [21, 65, 66]. Second, the species of Engelhardioideae subfamily were closely related and were divided into two main clades, which is consistent with the Sect. *Engelhardia* (Clade I) and Sect. *Psilocarpeae* (Clade II) [67] and get strongly supported (BS = 100%, PP = 1). The Clade I included *E. spicata*, *E. spicata* var. *rigida*, *E. hainanensis*, *E. serrata*, *E. anminiana*, and *E. villosa*. The clade II included *E. roxburghiana* and *E. fenzelii*, which were sister species. Third, *R. chiliantha*, the only species in Rhoipteleoideae subfamily, was located at the base of Juglandaceae in phylogenetic relationships and was also an endangered endemic species in China [3, 9, 68, 69].

### Exploring the origin and evolutionary relationship of Juglandaceae

In previous studies, the crown age of Juglandaceae based on fossil data was approximately 84 Mya during the Cretaceous [70, 71]. Our results indicated that the divergence time of Juglandaceae was approximately 97.69 Mya (95% HPD: 95.49–100.58 Mya) using an older fossil time node from a fossil plant *Budvaricarpus serialis* (ca. 85 Mya) [72, 73]. The three subfamilies Rhoipteleoideae, Engelhardioideae, and Juglandoideae, successively differentiated at 89.28 Mya (95% HPD: 85.61–92.96 Mya) and 73.59 Mya (95% HPD: 69.01–78.13 Mya) (Fig. 9).

The Juglandoideae subfamily differentiated approximately between 69.01 and 78.13 Mya, from Cretaceous to Paleogene. The Northern Tropical Hypothesis [74, 75] provides a reasonable explanation for the origin and diversity of the Juglandoideae subfamily, that is during the warm Paleocene and Eocene periods, species of the Juglandoideae subfamily formed and rapidly diversified, spreading from North America to Europe and Asia through the North Atlantic Road Bridge and the Bering Land Bridge [76]. However, the global cooling that occurred after the extreme heat period of the Paleocene Eocene led to the extinction of most species [77–79]. The *Cylocarya* and *Platycarya* were endemic to East Asia, while *Pterocarya* were mainly distributed in the Caucasus and East Asia regions of southern Russia. *Carya* and *Juglans* have a wide distribution range in Eurasia, possibly due to their nutty fruit morphology, which facilitates animal transportation and transmission [3]. According to our results, the divergence time of *Carya* and *Juglans* was about 64.98 Mya, of *Juglans* and *Pterocarya* was about 46.29 Mya, of *Pterocarya* and *Cyclocarya* was about 54.10 Mya. Therefore, we inferred that differentiation events within the Juglandoideae subfamily occurred a long time ago and had undergone a long evolutionary process [21, 66].

The divergence time of the two clades in the Engelhardioideae subfamily was approximately between 27.64 and 46.11 Mya, mainly occurring from the Early Eocene to the Middle Oligocene. The earliest fossil record of the fruit of *Engelhardia* existed in South America and North America, and the oldest *Alatonucula ignis* fossil had been found in the early Eocene strata of Argentina [65]. At the same time, a fossil was found in the Miocene strata of Alaska, USA (*Palaeocarya olsoni*) [70]. This means that these taxonomic groups were widely present in parts of the Northern and Southern Hemispheres during the Eocene. Perhaps due to the high temperatures in the Paleogene, the species of *Engelhardia* were widely distributed in high latitude areas. Based on the discovery of the earliest fruit fossil of *Palaeocarya* in China in the Late Eocene strata of Hainan Island (*Palaeocarya* sp.) [80], it indicates that *Engelhardia* plants began to occupy tropical Asia in the late Eocene, while species diversity emerged in the Oligocene Miocene.

In short, our study accurately estimated the divergence time of Juglandaceae species using 80 coding sequences (CDs) from plastomes. And we found that Juglandaceae species had a complex evolutionary history and species diversity, which may be influenced by geographical changes, climate changes, and animal coevolution during the evolutionary process.

## Conclusion

This study analyzed characteristics of the plastid genome of newly sequenced species of eight *Engelhardia* species, and clarified that the basic structure of the plastid genome was a typical tetrad structure. Three mutation hotspot regions were found and they can be used as potential molecular markers for inferring phylogenetic analysis and species identification. InDels may be an important driving factor for the plastome evolution of Juglandoideae and Engelhardioideae. Natural selection and mutation jointly affected the codon usage patterns of Juglandaceae and three subfamilies, with mutation pressure playing a major role. Phylogenetic results fully supported *Engelhardia* as a monophyletic group including two sects and the division of Juglandaceae into three subfamilies. Divergence time analysis revealed that *Engelhardia* originated in the later Cretaceous and diversified in the later Eocene, and Juglandaceae originated in the Early Cretaceous and differentiated in Middle Cretaceous. Overall, this study demonstrated that the plastome sequences displayed variable information to resolve phylogenetic relationships and were helpful to understand how species adapts to diverse ecological habitats.

## Materials and methods

### Plant materials and DNA extraction

In this work, a total of 13 individuals from all currently recognized eight species of *Engelhardia* (*E. anminiana*, *E. fenzelii*, *E. hainanensis*, *E. roxburghiana*, *E. serrata*, *E. spicata*, *E. spicata* var. *rigida*, and *E. villosa*) and one outgroup species *Rhoiptelea chiliantha* were collected in the tropical and subtropical Asia. The materials were identified by Yong-Hua Zhang, Hong-Hu Meng and Pan Li. The fresh leaves from each accession were dried with silica gel for further DNA extraction. Total high-quality genomic DNA was extracted from all plant materials using Plant DNAzol Reagent (Hangzhou Lifefeng Biotechnology Co., Ltd, Hangzhou, China). The detailed information of taxon, voucher number, collector and GenBank accession number are listed in Table 1.

### DNA resequencing, plastomes assembly and gene annotation

High-quality genomic DNA from each sample was used for the whole genome sequencing (WGS) to obtain paired-end 100 bp raw reads on the BGISEQ-500 platform (BGI, Shenzhen, China) according to the manufacturers’ procedures. The quality of raw reads was controlled by removing the Phred score lower than 30, remaining the high-quality sequences for genome assembly using the GetOrganelle software [81]. The command lines used for the assembly were as follows: get_organelle_reads.py -1 forward.fq -2 reverse.fq -o plastome_output -R 15 -k 21,45,65,85,105 -F plant_cp. All targeted plastomes sequences were concatenated and manually edited with Geneious Prime 2021 software (http://www.geneious.com/), using the plastome sequences of *Carya sinensis* (MN892516) and *Rhoiptelea chiliantha* (MT701585) as the reference genomes. At the same time, the CPGAVAS2 web server (http://www.herbalgenomics.org/cpgavas) was used to predict the types and structures of all the protein-coding and non-coding genes in the plastomes. The final plastomes annotation was determined by comparing the results of Geneious Prime 2021 and CPGAVAS2. Finally, CPGView [82] were used to visualize the plastome map. The 13 newly generated complete plastome sequences were deposited in GeneBank (Accession numbers were listed in Table 1). Plastomes of 43 other species were downloaded from NCBI GenBank repository and re-annotated using the earlier method, the GenBank accession numbers are shown in Table S1.

### Comparative analysis of plastome structure features

We used these newly-sequenced *Engelhardia* individuals to study the genomic variation in *Engelhardia*. Two methods were used for comparative genomic analysis: (1) The comparison of the plastomes sequence identity was using MAVUE and mVISTA [83]. Sequence rearrangement detection was performed on 13 plastomes using the Mauve alignment plugin in Geneious Prime 2021 software, and 13 sequences were aligned using the LAGAN model in the online software mVISTA. (2) The comparison of the expansion and contraction of IR regions was presented. The IR boundary regions were visualized by using the online website IRScope (https://irscope.shinyapps.io/irapp/).

### Repeat sequences detection

The genome of organisms, especially higher organisms, contains a large number of repeat sequences, which can be divided into dispersed repeat sequence (DRS) and tandem repeat sequence [84] according to their distribution patterns in the genome. First, the DRS in the plastomes of eight *Engelhardia* species were predicted by the REPuter software [85]. The forward, reverse, palindromic and complement repeat sequences were identified using the following parameters: length of repeat unit ≥ 30 bp, sequence consistency ≥ 90% (Hamming distance = 3). Then, the TRS in the plastomes were predicted by using the Tandom Repeats Finder (TRF) web server (https://tandem.bu.edu/trf/trf.html). Finally, the MISA software was used to identify simple sequence repeats (SSR), setting the minimum repetition threshold values for mono-, di-, tri-, tetra-, penta-, and hexa-nucleotide were set to 10, 5, 4, 3, 3, 3, respectively.

### Analysis of nucleotide polymorphism and mutation sites

We analysed nucleotide polymorphisms based on the Pi values of *Carya*, *Engelhardia*, and *Juglans*, respectively. The plastomes were aligned using mafft alignment with default settings in Geneious Prime 2021. The Pi of protein-coding genes, noncoding genes, and the intergenic regions extracted from the plastome was calculated using DnaSP v6.0 [86] to show the nucleotide diversity at genus level. In order to eliminate interference from different individuals of the same species, we only chosen *E. hainanensis*_HN01, *E. fenzelii*_TTD01 and *E. roxburghiana*_BPZ11 accessions on behalf of *E. hainanensis*, *E. fenzelii*, and *E. roxburghiana*, respectively. The parameters were set as: window length = 600 bp, step size = 200 bp. After that, the corresponding loci were located and counted in Generous Prime 2021 software, and sequence fragments with Pi values greater than 0.01 were used as candidate high variation regions.

In order to conduct comprehensive comparison of genomic variation of *Carya*, *Engelhardia*, and *Juglans* plastomes, we calculated their total number, length, and percentage of SNVs and insertion and deletions (InDels) sites located in gene intervals, exons, introns, and RNA genes. For *Engelhardia* species, we only kept one individual from the same species to conduct nucleotide polymorphisms analysis. To draw density bar graphs of SNVs, InDels data, we used the Genome Varscan plugin in TBtools, and the detection parameters were set as: the number of threads (CPU) to 2, the genome sequence divergence standard (Diff) to OneIn Thousand, VarRange from 0 to 1,000,000. *Carya*, *Engelhardia*, and *Juglans* were aligned with *R. chiliantha*, which was selected as the reference sequence, then variation site information was outputted.

### Codon usage bias

Different species use different codons with different frequencies, and there will be certain preferences [87, 88]. Studying the differences in codon usage patterns among families or genera can help us effectively understand the genetic evolution patterns of species. In addition, exploring the codon usage patterns of plant plastomes is beneficial to explore the adaptive mechanisms of plants under different evolutionary patterns [45]. In addition to the overall analysis of Juglandaceae, we also analyzed Engelhardioideae, Juglandoideae, and Rhoipteleoideae. The consensus genes encoding proteins with a sequence length longer than 300 bp and the start codon of ATG were used to analyse codon bias. T3s, C3s, A3s, G3s, CAI, CBI, Fop, ENC and GC values were calculated using CodonW software (http://codonw.sourceforge.net/). And plot according to the calculated correlation values: (I) Plot with ENC as the y-axis and GC3s as the x-axis to evaluate the influence of base composition on codon usage bias, the observed ENC value was compared with the expected ENC value using the following equation: ENC = 2 + GC3s + 29/[GC3s2 + (1—GC3s)2]; (II) Using [A3/(A3 + T3)] as the y-axis, with [G3/(G3 + C3)] as the x-axis, draw a coordinate map to assess the effects of genetic mutation and natural selection on codon usage preferences. All the screened genes were sequenced according to ENC value as a whole. The upper and lower 5% gene samples were selected and defined as low expression group and high expression group, and the RSCU value of each group was calculated. The RSCU difference between low expression group and high expression group was calculated. The codon with RSCU > 1 and △RSCU > 0.08 is defined as the optimal codon.

### Phylogenetic relationships of Juglandaceae

Plastomes were aligned using MAFFT v 7.308 [89] implemented in Geneious Prime 2021. Maximum Likelihood (ML) analysis and Bayesian Inference (BI) were employed for the phylogenomic reconstruction of Juglandaceae. The best-fit nucleotide substation model for ML and BI analysis was determined by Modeltest v3.7 [90], in which the complete plastomes data was GTR + I + G and BI analyses were performed using the RAxML-HPC v8.1.11 and MrBayes v3.2.3 online tools available from the CIPRES Science Gateway web server [91, 92]. The ML analysis was conducted with default settings with 1000 bootstrap replicates. BI trees were produced with the setting of 5,000,000 generations, under GTRGAMMA model with one cold and three incrementally heated Markov Chain Monte Carlo (MCMC) run simultaneously [92] in two parallel runs sampling every 1000 generations. The first 25% of the trees were discarded as burn-in. The remaining trees were used for generating the consensus tree.

### Evolutionary analysis of Juglandaceae plastomes

We observed *Ks* (synonymous), *Ka* (non-synonymous) substitution, and *Ka/Ks* ratio using pairwise alignment of protein-coding sequences of *Morella rubra* and other 50 selected species of Juglandaceae. We used *M. rubra* as the reference in each pair of alignments to make pairwise alignments with every gene. Geneious Prime 2021 was used to extract 80 common protein-coding genes, and DnaSP v6.0 was used to calculate the *Ka* and *Ks* substitutions. In addition, in order to detect the selection pressure on whole plastid genes with different functions, CDS genes were divided into photosynthesis-related, self-replication-related, and other functional genes (Table S1). Finally, we plotted boxplot graphs of the *Ka/Ks* values of CDS genes based on different functional classifications or taxonomic groups, and marked the significance of the differences between the groups. All analyses were conducted in R version 4.3.0 (https://www.R-project.org/).

### Divergence-time estimation and fossil calibration

We estimated the divergence time of Juglandaceae species based on 80 coding sequences (CDSs), combined with the calibration of four fossils (Table S8) [24, 70–73, 93–96]. The nucleotide substitution model is the same as the MrBayes parameters mentioned above. Before setting to Yule Process Specialty Tree model, set the molecular clock to log normal relaxation Molecular clock. For the MCMC program, the chain length was 5 × 10^8^ generations, sampling every 10,000 generations. All options were set in BEAUTi v1.10.4, exported as an XML file, and run in BEAST v1.10.4 [97]. We checked the convergence of the Markov chains in Tracer v.1.6 (http://beast.bio.ed.ac.uk/Tracer/) and combined the chains after removing a burn-in of the first 50% generations. The effective sample sizes (ESS) exceeded 200 for all parameters. The program FigTree v1.4.3 (http://tree.bio.ed.ac.uk/software/figtree/) was used to visualize mean node ages and highest posterior density (HPD) intervals at 95% (upper and lower) for each node and to estimate branch lengths and divergence times.

### Supplementary Information

Supplementary Material 1.

Supplementary Material 2.

## Data Availability

Sequence data that support the findings of this study have been deposited in the China National GeneBank DataBase under accession number CNP0005522. The voucher number and collector of the species are as follow, Engelhardia anminiana (MHH2018001-02, Hong-Hu Meng), E. fenzelii_JNSX01 (ZYH19072801, Yong-Hua Zhang), E. fenzelii_TTD01 (ZYH17102801, Yong-Hua Zhang), E. hainanensis_02 (MHH20170514001A, Hong-Hu Meng), E. hainanensis_HN01 (ZYH18072101, Yong-Hua Zhang), E. roxburghiana_BPZ11 (ZYH17120911, Yong-Hua Zhang), E. roxburghiana_JFL02 (ZYH18072103, Yong-Hua Zhang), E. roxburghiana_TPS06 (ZYH17121606, Yong-Hua Zhang), E. roxburghiana_XSBN01 (ZYH19011503, Yong-Hua Zhang), E. serrata (MHH201800103-10, Hong-Hu Meng), E. spicata (MHH2018092101-01, Hong-Hu Meng), E. spicata var. rigida (MHH20180922015-16, Hong-Hu Meng), E. villosa (MHH2018032813-20, Hong-Hu Meng), Rhoiptelea chiliantha_MWS2 (LP174627, Pan Li).
