# Supplementary material for: Plastome evolution of Engelhardia facilitates phylogeny of Juglandaceae
Source: BMC Plant Biol. 2024 Jul 6;24:634. doi: 10.1186/s12870-024-05293-0 (PMC11227234; doi:10.1186/s12870-024-05293-0)
Supplement: Supplementary file 1 — Supplementary Material 1. [file 12870_2024_5293_MOESM1_ESM.zip › Supplementary table/Table S1.docx]

**Table S1** The species name, NCBI number, family, and subfamily of all species involved in the study.

| **Species** | **GenBank** | **Family** | **Subfamily** |
| --- | --- | --- | --- |
| *Carya aquatica* | MW255965 | Juglandaceae | Juglandoideae |
| *Carya cathayensis* | MN892516 | Juglandaceae | Juglandoideae |
| *Carya cordiformis* | MW368387 | Juglandaceae | Juglandoideae |
| *Carya floridana* | MW410229 | Juglandaceae | Juglandoideae |
| *Carya glabra* | MW410230 | Juglandaceae | Juglandoideae |
| *Carya hunanensis* | MH188303 | Juglandaceae | Juglandoideae |
| *Carya illinoinensis* | MH188302 | Juglandaceae | Juglandoideae |
| *Carya kweichowensis* | MH188301 | Juglandaceae | Juglandoideae |
| *Carya laciniosa* | MW186783 | Juglandaceae | Juglandoideae |
| *Carya myristiciformis* | ON584556 | Juglandaceae | Juglandoideae |
| *Carya ovalis* | MW440674 | Juglandaceae | Juglandoideae |
| *Carya ovata* | MW410233 | Juglandaceae | Juglandoideae |
| *Carya palmeri* | MW410234 | Juglandaceae | Juglandoideae |
| *Carya poilanei* | ON568300 | Juglandaceae | Juglandoideae |
| *Carya sinensis* | MW421595 | Juglandaceae | Juglandoideae |
| *Carya texana* | MW410235 | Juglandaceae | Juglandoideae |
| *Carya tomentosa* | MW410236 | Juglandaceae | Juglandoideae |
| *Carya tonkinensis* | MW368388 | Juglandaceae | Juglandoideae |
| *Cyclocarya paliurus* | NC_034315 | Juglandaceae | Juglandoideae |
| *Juglans ailanthifolia* | MH188299 | Juglandaceae | Juglandoideae |
| *Juglans cinerea* | MH188298 | Juglandaceae | Juglandoideae |
| *Juglans hindsii* | MH188297 | Juglandaceae | Juglandoideae |
| *Juglans hopeiensis* | KX671977 | Juglandaceae | Juglandoideae |
| *Juglans major* | MH188296 | Juglandaceae | Juglandoideae |
| *Juglans mandshurica* | KX671975 | Juglandaceae | Juglandoideae |
| *Juglans microcarpa* | MH188295 | Juglandaceae | Juglandoideae |
| *Juglans nigra* | MH188294 | Juglandaceae | Juglandoideae |
| *Juglans regia* | MN397935 | Juglandaceae | Juglandoideae |
| *Juglans sigillata* | KX424843 | Juglandaceae | Juglandoideae |
| *Platycarya strobilacea* | NC_035413 | Juglandaceae | Juglandoideae |
| *Pterocarya fraxinifolia* | MH188291 | Juglandaceae | Juglandoideae |
| *Pterocarya hupehensis* | MH188293 | Juglandaceae | Juglandoideae |
| *Pterocarya macroptera* var. *insignis* | MH188292 | Juglandaceae | Juglandoideae |
| *Pterocarya stenoptera* | MH188289 | Juglandaceae | Juglandoideae |
| *Pterocarya tonkinensis* | MH188288 | Juglandaceae | Juglandoideae |
| *Engelhardia anminiana* | OR208248 | Juglandaceae | Engelhardioideae |
| *Engelhardia fenzelii*_JNSX01 | OP480035 | Juglandaceae | Engelhardioideae |
| *Engelhardia fenzelii*_TTD01 | OP480037 | Juglandaceae | Engelhardioideae |
| *Engelhardia hainanensis*_02 | OR208247 | Juglandaceae | Engelhardioideae |
| *Engelhardia hainanensis_*HN01 | OP480038 | Juglandaceae | Engelhardioideae |
| *Engelhardia roxburghiana*_BPZ11 | OP480042 | Juglandaceae | Engelhardioideae |
| *Engelhardia roxburghiana*_JFL02 | OP480031 | Juglandaceae | Engelhardioideae |
| *Engelhardia roxburghiana*_TPS06 | OP480033 | Juglandaceae | Engelhardioideae |
| *Engelhardia roxburghiana*_XSBN01 | OP480034 | Juglandaceae | Engelhardioideae |
| *Engelhardia serrata* | OR208250 | Juglandaceae | Engelhardioideae |
| *Engelhardia spicata* | OR208253 | Juglandaceae | Engelhardioideae |
| *Engelhardia spicata* var. *rigida* | OR208251 | Juglandaceae | Engelhardioideae |
| *Engelhardia villosa* | OR208252 | Juglandaceae | Engelhardioideae |
| *Rhoiptelea chiliantha*_LSX | PP496514 | Juglandaceae | Rhoipteleoideae |
| *Rhoiptelea chiliantha*_MWS2 | OP480039 | Juglandaceae | Rhoipteleoideae |
| *Mycirca rubra/Morella rubra* | NC_035006 | Myricaceae | / |
| *Carpinus putoensis* | NC_033503 | Betulaceae | / |
| *Ostrya rehderiana* | NC_028349 | Betulaceae | / |
| *Corylus chinensis* | KX814336 | Betulaceae | / |
| *Betula nana* | MT872530 | Betulaceae | / |
| *Quercus rubra* | MK105463 | Fagaceae | / |
