# Supplementary material for: Plastome evolution of Engelhardia facilitates phylogeny of Juglandaceae
Source: BMC Plant Biol. 2024 Jul 6;24:634. doi: 10.1186/s12870-024-05293-0 (PMC11227234; doi:10.1186/s12870-024-05293-0)
Supplement: Supplementary file 1 — Supplementary Material 1. [file 12870_2024_5293_MOESM1_ESM.zip › Supplementary table/Table S2.docx]

**Table** **S2** Genes contents in the plastomes of *Engelhardia* species*.*

| **Gene category** | **Groups** | **Name** |
| --- | --- | --- |
| Self-replication | Transfer RNA | *trnA*-*UGC*^a*,b^(×2), *trnC*-*GCA*, *trnD*-*GUC*, *trnE*-*UUC*, *trnF*-*GAA*, *trnG*-*UCC*^a*^, *trnG*-*UCC*, *trnH*-*GUG*, *trnI*-*CAU*^b^(×2), *trnI*-*GAU*^a*,b^(×2), *trnK*-*UUU*^a*^, *trnL*-*CAA*^b^(×2), *trnL*-*UAA*^a*^, *trnL*-*UAG*, *trnM*-*CAU*(×2), *trnN*-*GUU*^b^(×2), *trnP*-*UGG*, *trnQ*-*UUG*, *trnR*-*ACG*^b^(×2), *trnR*-*UCU*, *trnS*-*GCU*, *trnS*-*GGA*, *trnS*-*UGA*, *trnT*-*GGU*, *trnT*-*UGU*, *trnV*-*GAC*^b^(×2), *trnV*-*UAC*^a*^, *trnW*-*CCA*, *trnY*-*GUA* |
|  | Ribosome RNA | *rrn4.5*^b^(×2), *rrn5*^b^(×2), *rrn16*^b^(×2), *rrn23*^b^(×2) |
|  | Large subunit | *rpl2*^a*,b^(×2) , *rpl14*, *rpl16*^a*^, *rpl20*, *rpl22*, *rpl23*^b^(×2), *rpl32*, *rpl33*, *rpl36* |
|  | Small subunit | *rps2*, *rps3*, *rps4*, *rps7*^b^(×2), *rps8*, *rps11*, *rps12*^a**,b^(×2), *rps14*, *rps15*, *rps16*^a*^, *rps18*, *rps19*, *Ψrps19*^b^ (a short one in all *Engelhardia*) |
|  | RNA polymerase | *rpo*A, *rpo*B, *rpo*C1^a*^, *rpo*C2 |
| Photosynthesis | ATP synthase | *atp*A, *atp*B, *atp*E, *atp*F^a*^, *atp*H, *atp*I |
|  | NADH dehydrogenase | *ndhA*^a*^, *ndhB*^a*,b^(×2), *ndhC*, *ndhD*, *ndhE*, *ndhF*, *ndhG*, *ndhH*, *ndhI*, *ndhJ*, *ndhK* |
|  | Cytochrome | *petA*, *petB*^a*^, *petD*^a*^, *petG*, *petL*, *petN* |
|  | Photosystem I | *psaA*, *psaB*, *psaC*, *psaI*, *psaJ* |
|  | Photosystem II | *psbA*, *psbB*, *psbC*, *psbD*, *psbE*, *psbF*, *psbH*, *psbI*, *psbJ*, *psbK*, *psbL*, *psbM*, *psbN*, *psbT*, *psbZ* |
| Other genes | Rubisco | *rbcL* |
|  | Acetyl-CoA carboxylase | *accD* |
|  | C-Type cytochrome synthesis | *ccsA* |
|  | Envelop membrane protein | *cemA* |
|  | Maturase | *matK* |
|  | Protease | *clpP*^a**^ |
|  | Hypothetical reading frames | *ycf1*^b^, *Ψycf1*^b^ (a short one in all *Engelhardia*) ,*ycf2*^b^(×2), *ycf3* ^a**^, *ycf4*, *ycf15*^b^(×2) |
|  | Translation initiation factor | *infA* |

a, genes containing introns; superscript *, the number of introns in the gene; b, genes located in IR regions, ×2, genes with two copies.
