# Supplementary material for: Plastome evolution of Engelhardia facilitates phylogeny of Juglandaceae
Source: BMC Plant Biol. 2024 Jul 6;24:634. doi: 10.1186/s12870-024-05293-0 (PMC11227234; doi:10.1186/s12870-024-05293-0)
Supplement: Supplementary file 1 — Supplementary Material 1. [file 12870_2024_5293_MOESM1_ESM.zip › Supplementary table/Table S3.docx]

**Table S3** The GC content of each part of plastomes of *Engelhardia* species and *Rhoiptelea chiliantha*

| **Species** | **GC content of whole genome** | **GC content of LSC** | **GC content**  **of SSC** | | **GC content**  **of IRs** | **GC content of CDS** |
| --- | --- | --- | --- | --- | --- | --- |
| *E. anminiana* | 35.9 | 33.4 | 29.6 | 42.7 | | 37.2 |
| *E. fenzelii*_JNSX01 | 36.0 | 33.6 | 29.4 | 42.6 | | 37.3 |
| *E. fenzelii*_TTD01 | 36.0 | 33.5 | 29.4 | 42.6 | | 37.3 |
| *E. hainanensis*_02 | 35.8 | 33.2 | 29.5 | 42.6 | | 37.2 |
| *E. hainanensis*_HN01 | 35.8 | 33.2 | 29.5 | 42.6 | | 37.2 |
| *E. roxburghiana*_BPZ11 | 35.9 | 33.5 | 29.3 | 42.6 | | 37.2 |
| *E. roxburghiana*_JFL02 | 35.9 | 33.5 | 29.3 | 42.6 | | 37.2 |
| *E. roxburghiana*_TPS06 | 35.9 | 33.5 | 29.3 | 42.6 | | 37.2 |
| *E. roxburghiana*_XSBN01 | 35.9 | 33.5 | 29.3 | 42.6 | | 37.2 |
| *E. serrata* | 35.9 | 33.4 | 29.5 | 42.7 | | 37.3 |
| *E. spicata* | 35.8 | 33.3 | 29.6 | 42.6 | | 37.2 |
| *E. spicata* var. *rigida* | 35.9 | 33.3 | 29.6 | 42.6 | | 37.2 |
| *E. villosa* | 35.8 | 33.2 | 29.6 | 42.6 | | 37.3 |
| *R. chiliantha*_MWS2 | 36.1 | 33.6 | 29.8 | 42.5 | | 37.4 |

LSC: large single copy region; SSC: small single copy region; IR: inverted repeats; CDS: protein-coding genes
