# Supplementary material for: Plastome evolution of Engelhardia facilitates phylogeny of Juglandaceae
Source: BMC Plant Biol. 2024 Jul 6;24:634. doi: 10.1186/s12870-024-05293-0 (PMC11227234; doi:10.1186/s12870-024-05293-0)
Supplement: Supplementary file 1 — Supplementary Material 1. [file 12870_2024_5293_MOESM1_ESM.zip › Supplementary table/Table S5.docx]

**Table S5a** Statistic of genomic variation events detected in the LSC, SSC and IR regions across the Juglandaceae plastomes using *R. chiliantha* as outgroup.

| **Species** | **SNV(**single nucleotide variants**)** | | | | **Deletion** | | | | **Insertion** | | | |
| --- | --- | --- | --- | --- | --- | --- | --- | --- | --- | --- | --- | --- |
|  | **LSC** | **SSC** | **IR** | **Total** | **LSC** | **SSC** | **IR** | **Total** | **LSC** | **SSC** | **IR** | **Total** |
| *Carya aquatica* | 884 | 702 | 317 | 1903 | 100 | 36 | 22 | 158 | 88 | 35 | 19 | 142 |
| *Carya cathayensis* | 1228 | 713 | 281 | 2222 | 148 | 37 | 24 | 209 | 138 | 36 | 18 | 192 |
| *Carya cordiformis* | 883 | 703 | 317 | 1903 | 100 | 35 | 22 | 157 | 89 | 35 | 19 | 143 |
| *Carya floridana* | 884 | 704 | 317 | 1905 | 100 | 35 | 22 | 157 | 88 | 35 | 19 | 142 |
| *Carya glabra* | 888 | 693 | 311 | 1892 | 101 | 36 | 25 | 162 | 86 | 35 | 17 | 138 |
| *Carya hunanensis* | 1095 | 0 | 140 | 1235 | 124 | 0 | 11 | 135 | 124 | 0 | 8 | 132 |
| *Carya illinoinensis* | 883 | 697 | 300 | 1880 | 100 | 36 | 22 | 158 | 89 | 35 | 15 | 139 |
| *Carya kweichowensis* | 1909 | 0 | 140 | 2049 | 225 | 0 | 11 | 236 | 190 | 0 | 9 | 199 |
| *Carya laciniosa* | 884 | 698 | 326 | 1908 | 100 | 36 | 21 | 157 | 88 | 35 | 20 | 143 |
| *Carya myristiciformis* | 888 | 699 | 320 | 1907 | 99 | 36 | 23 | 158 | 88 | 36 | 19 | 143 |
| *Carya ovalis* | 883 | 700 | 342 | 1925 | 100 | 37 | 25 | 162 | 89 | 36 | 21 | 146 |
| *Carya ovata* | 883 | 701 | 319 | 1903 | 101 | 35 | 22 | 158 | 88 | 36 | 17 | 141 |
| *Carya palmeri* | 901 | 712 | 308 | 1921 | 101 | 36 | 25 | 162 | 91 | 35 | 17 | 143 |
| *Carya poilanei* | 859 | 671 | 267 | 1797 | 98 | 40 | 18 | 156 | 91 | 30 | 17 | 138 |
| *Carya sinensis* | 1174 | 674 | 309 | 2157 | 130 | 38 | 21 | 189 | 134 | 33 | 15 | 182 |
| *Carya texana* | 885 | 693 | 310 | 1888 | 102 | 36 | 23 | 161 | 87 | 35 | 17 | 139 |
| *Carya tomentosa* | 882 | 704 | 317 | 1903 | 100 | 35 | 22 | 157 | 89 | 35 | 19 | 143 |
| *Carya tonkinensis* | 864 | 670 | 266 | 1800 | 99 | 40 | 18 | 157 | 89 | 30 | 17 | 136 |
| *Cyclocarya_paliurus* | 1947 | 679 | 254 | 2880 | 203 | 37 | 19 | 259 | 187 | 35 | 7 | 229 |
| *Engelhardia anminiana* | 1269 | 865 | 278 | 2412 | 128 | 47 | 33 | 208 | 147 | 47 | 14 | 208 |
| *Engelhardia fenzelii*_JNSX01 | 2022 | 800 | 261 | 3083 | 197 | 47 | 30 | 274 | 227 | 50 | 20 | 297 |
| *Engelhardia fenzelii*_TTD01 | 2020 | 797 | 261 | 3078 | 196 | 47 | 29 | 272 | 228 | 50 | 20 | 298 |
| *Engelhardia hainanensis*_02 | 975 | 817 | 247 | 2039 | 91 | 34 | 30 | 155 | 125 | 51 | 11 | 187 |
| *Engelhardia hainanensis*_HN01 | 975 | 819 | 247 | 2041 | 91 | 34 | 30 | 155 | 125 | 50 | 11 | 186 |
| *Engelhardia roxburghiana*_BPZ11 | 1955 | 807 | 254 | 3016 | 187 | 48 | 29 | 264 | 221 | 50 | 20 | 291 |
| *Engelhardia roxburghiana*_JFL02 | 1933 | 805 | 250 | 2988 | 189 | 45 | 29 | 263 | 224 | 50 | 20 | 294 |
| *Engelhardia roxburghiana*_TPS06 | 1955 | 807 | 254 | 3016 | 187 | 48 | 29 | 264 | 221 | 50 | 20 | 291 |
| *Engelhardia roxburghiana*_XSBN01 | 1951 | 806 | 254 | 3011 | 187 | 48 | 29 | 264 | 219 | 50 | 20 | 289 |
| *Engelhardia serrata* | 1296 | 860 | 285 | 2441 | 116 | 49 | 33 | 198 | 150 | 49 | 14 | 213 |
| *Engelhardia spicata* | 2403 | 837 | 264 | 3504 | 230 | 42 | 34 | 306 | 267 | 50 | 12 | 329 |
| *Engelhardia spicata* var. *rigida* | 1170 | 809 | 259 | 2238 | 106 | 42 | 37 | 185 | 143 | 49 | 13 | 205 |
| *Engelhardia villosa* | 2506 | 864 | 280 | 3650 | 237 | 43 | 31 | 311 | 295 | 51 | 18 | 364 |
| *Juglans ailanthifolia* | 1160 | 671 | 271 | 2102 | 130 | 41 | 21 | 192 | 112 | 30 | 7 | 149 |
| *Juglans cinerea* | 1937 | 688 | 280 | 2905 | 215 | 40 | 20 | 275 | 181 | 29 | 9 | 219 |
| *Juglans hindsii* | 1752 | 659 | 296 | 2707 | 250 | 49 | 25 | 324 | 166 | 31 | 11 | 208 |
| *Juglans hopeiensis* | 1925 | 668 | 263 | 2856 | 212 | 39 | 21 | 272 | 170 | 30 | 7 | 207 |
| *Juglans major* | 1797 | 670 | 268 | 2735 | 183 | 41 | 20 | 244 | 171 | 30 | 9 | 210 |
| *Juglans mandshurica* | 1926 | 667 | 268 | 2861 | 211 | 37 | 21 | 269 | 170 | 32 | 7 | 209 |
| *Juglans microcarpa* | 1769 | 675 | 268 | 2712 | 248 | 45 | 38 | 331 | 148 | 28 | 10 | 186 |
| *Juglans nigra* | 1952 | 672 | 268 | 2892 | 209 | 42 | 20 | 271 | 190 | 31 | 9 | 230 |
| *Juglans regia* | 1933 | 678 | 271 | 2882 | 212 | 41 | 19 | 272 | 177 | 28 | 7 | 212 |
| *Juglans sigillata* | 1928 | 676 | 270 | 2874 | 212 | 41 | 19 | 272 | 177 | 28 | 7 | 212 |
| *Platycarya strobilacea* | 649 | 855 | 389 | 1893 | 58 | 55 | 34 | 147 | 60 | 44 | 49 | 153 |
| *Pterocarya fraxinifolia* | 1958 | 672 | 263 | 2893 | 218 | 39 | 22 | 279 | 186 | 31 | 12 | 229 |
| *Pterocarya hupehensis* | 1946 | 693 | 268 | 2907 | 221 | 41 | 22 | 284 | 167 | 33 | 14 | 214 |
| *Pterocarya macroptera var. insignis* | 1940 | 664 | 260 | 2864 | 215 | 39 | 23 | 277 | 182 | 32 | 9 | 223 |
| *Pterocarya stenoptera* | 650 | 668 | 268 | 1586 | 70 | 38 | 21 | 129 | 54 | 29 | 9 | 92 |

**Table S5b** Statistic of genomic variation events of every 1 Kb detected in the LSC, SSC and IR regions across the Juglandaceae plastomes using *R. chiliantha* as outgroup.

| **Species** | **SNV(**single nucleotide variants**)** | | | | **Deletion** | | | | **Insertion** | | | |
| --- | --- | --- | --- | --- | --- | --- | --- | --- | --- | --- | --- | --- |
|  | **LSC** | **SSC** | **IR** | **Total** | **LSC** | **SSC** | **IR** | **Total** | **LSC** | **SSC** | **IR** | **Total** |
| *Carya aquatica* | 5.53 | 4.39 | 1.98 | 11.89 | 0.63 | 0.23 | 0.14 | 0.99 | 0.55 | 0.22 | 0.12 | 0.89 |
| *Carya cathayensis* | 7.68 | 4.46 | 1.76 | 13.89 | 0.93 | 0.23 | 0.15 | 1.31 | 0.86 | 0.23 | 0.11 | 1.20 |
| *Carya cordiformis* | 5.52 | 4.39 | 1.98 | 11.89 | 0.63 | 0.22 | 0.14 | 0.98 | 0.56 | 0.22 | 0.12 | 0.89 |
| *Carya floridana* | 5.53 | 4.40 | 1.98 | 11.91 | 0.63 | 0.22 | 0.14 | 0.98 | 0.55 | 0.22 | 0.12 | 0.89 |
| *Carya glabra* | 5.55 | 4.33 | 1.94 | 11.83 | 0.63 | 0.23 | 0.16 | 1.01 | 0.54 | 0.22 | 0.11 | 0.86 |
| *Carya hunanensis* | 6.84 | 0.00 | 0.88 | 7.72 | 0.78 | 0.00 | 0.07 | 0.84 | 0.78 | 0.00 | 0.05 | 0.83 |
| *Carya illinoinensis* | 5.52 | 4.36 | 1.88 | 11.75 | 0.63 | 0.23 | 0.14 | 0.99 | 0.56 | 0.22 | 0.09 | 0.87 |
| *Carya kweichowensis* | 11.93 | 0.00 | 0.88 | 12.81 | 1.41 | 0.00 | 0.07 | 1.48 | 1.19 | 0.00 | 0.06 | 1.24 |
| *Carya laciniosa* | 5.53 | 4.36 | 2.04 | 11.93 | 0.63 | 0.23 | 0.13 | 0.98 | 0.55 | 0.22 | 0.13 | 0.89 |
| *Carya myristiciformis* | 5.55 | 4.37 | 2.00 | 11.92 | 0.62 | 0.23 | 0.14 | 0.99 | 0.55 | 0.23 | 0.12 | 0.89 |
| *Carya ovalis* | 5.52 | 4.38 | 2.14 | 12.03 | 0.63 | 0.23 | 0.16 | 1.01 | 0.56 | 0.23 | 0.13 | 0.91 |
| *Carya ovata* | 5.52 | 4.38 | 1.99 | 11.89 | 0.63 | 0.22 | 0.14 | 0.99 | 0.55 | 0.23 | 0.11 | 0.88 |
| *Carya palmeri* | 5.63 | 4.45 | 1.93 | 12.01 | 0.63 | 0.23 | 0.16 | 1.01 | 0.57 | 0.22 | 0.11 | 0.89 |
| *Carya poilanei* | 5.37 | 4.19 | 1.67 | 11.23 | 0.61 | 0.25 | 0.11 | 0.98 | 0.57 | 0.19 | 0.11 | 0.86 |
| *Carya sinensis* | 7.34 | 4.21 | 1.93 | 13.48 | 0.81 | 0.24 | 0.13 | 1.18 | 0.84 | 0.21 | 0.09 | 1.14 |
| *Carya texana* | 5.53 | 4.33 | 1.94 | 11.80 | 0.64 | 0.23 | 0.14 | 1.01 | 0.54 | 0.22 | 0.11 | 0.87 |
| *Carya tomentosa* | 5.51 | 4.40 | 1.98 | 11.89 | 0.63 | 0.22 | 0.14 | 0.98 | 0.56 | 0.22 | 0.12 | 0.89 |
| *Carya tonkinensis* | 5.40 | 4.19 | 1.66 | 11.25 | 0.62 | 0.25 | 0.11 | 0.98 | 0.56 | 0.19 | 0.11 | 0.85 |
| *Carya* average | 6.17 | 3.87 | 1.81 | 11.84 | 0.70 | 0.20 | 0.13 | 1.04 | 0.63 | 0.19 | 0.11 | 0.93 |
| *Cyclocarya_paliurus* | 12.17 | 4.24 | 1.59 | 18.00 | 1.27 | 0.23 | 0.12 | 1.62 | 1.17 | 0.22 | 0.04 | 1.43 |
| *Engelhardia anminiana* | 7.93 | 5.41 | 1.74 | 15.08 | 0.80 | 0.29 | 0.21 | 1.30 | 0.92 | 0.29 | 0.09 | 1.30 |
| *Engelhardia fenzelii*_JNSX01 | 12.64 | 5.00 | 1.63 | 19.27 | 1.23 | 0.29 | 0.19 | 1.71 | 1.42 | 0.31 | 0.13 | 1.86 |
| *Engelhardia fenzelii*_TTD01 | 12.63 | 4.98 | 1.63 | 19.24 | 1.23 | 0.29 | 0.18 | 1.70 | 1.43 | 0.31 | 0.13 | 1.86 |
| *Engelhardia hainanensis*_02 | 6.09 | 5.11 | 1.54 | 12.74 | 0.57 | 0.21 | 0.19 | 0.97 | 0.78 | 0.32 | 0.07 | 1.17 |
| *Engelhardia hainanensis*_HN01 | 6.09 | 5.12 | 1.54 | 12.76 | 0.57 | 0.21 | 0.19 | 0.97 | 0.78 | 0.31 | 0.07 | 1.16 |
| *Engelhardia roxburghiana*_BPZ11 | 12.22 | 5.04 | 1.59 | 18.85 | 1.17 | 0.30 | 0.18 | 1.65 | 1.38 | 0.31 | 0.13 | 1.82 |
| *Engelhardia roxburghiana*_JFL02 | 12.08 | 5.03 | 1.56 | 18.68 | 1.18 | 0.28 | 0.18 | 1.64 | 1.40 | 0.31 | 0.13 | 1.84 |
| *Engelhardia roxburghiana*_TPS06 | 12.22 | 5.04 | 1.59 | 18.85 | 1.17 | 0.30 | 0.18 | 1.65 | 1.38 | 0.31 | 0.13 | 1.82 |
| *Engelhardia roxburghiana*_XSBN01 | 12.19 | 5.04 | 1.59 | 18.82 | 1.17 | 0.30 | 0.18 | 1.65 | 1.37 | 0.31 | 0.13 | 1.81 |
| *Engelhardia serrata* | 8.10 | 5.38 | 1.78 | 15.26 | 0.73 | 0.31 | 0.21 | 1.24 | 0.94 | 0.31 | 0.09 | 1.33 |
| *Engelhardia spicata* | 15.02 | 5.23 | 1.65 | 21.90 | 1.44 | 0.26 | 0.21 | 1.91 | 1.67 | 0.31 | 0.08 | 2.06 |
| *Engelhardia spicata* var. *rigida* | 7.31 | 5.06 | 1.62 | 13.99 | 0.66 | 0.26 | 0.23 | 1.16 | 0.89 | 0.31 | 0.08 | 1.28 |
| *Engelhardia villosa* | 15.66 | 5.40 | 1.75 | 22.81 | 1.48 | 0.27 | 0.19 | 1.94 | 1.84 | 0.32 | 0.11 | 2.28 |
| *Engelhardia* average | 10.62 | 5.20 | 1.66 | 17.48 | 1.01 | 0.28 | 0.20 | 1.48 | 1.23 | 0.31 | 0.10 | 1.64 |
| *Juglans ailanthifolia* | 7.25 | 4.19 | 1.69 | 13.14 | 0.81 | 0.26 | 0.13 | 1.20 | 0.70 | 0.19 | 0.04 | 0.93 |
| *Juglans cinerea* | 12.11 | 4.30 | 1.75 | 18.16 | 1.34 | 0.25 | 0.13 | 1.72 | 1.13 | 0.18 | 0.06 | 1.37 |
| *Juglans hindsii* | 10.95 | 4.12 | 1.85 | 16.92 | 1.56 | 0.31 | 0.16 | 2.03 | 1.04 | 0.19 | 0.07 | 1.30 |
| *Juglans hopeiensis* | 12.03 | 4.18 | 1.64 | 17.85 | 1.33 | 0.24 | 0.13 | 1.70 | 1.06 | 0.19 | 0.04 | 1.29 |
| *Juglans major* | 11.23 | 4.19 | 1.68 | 17.09 | 1.14 | 0.26 | 0.13 | 1.53 | 1.07 | 0.19 | 0.06 | 1.31 |
| *Juglans mandshurica* | 12.04 | 4.17 | 1.68 | 17.88 | 1.32 | 0.23 | 0.13 | 1.68 | 1.06 | 0.20 | 0.04 | 1.31 |
| *Juglans microcarpa* | 11.06 | 4.22 | 1.68 | 16.95 | 1.55 | 0.28 | 0.24 | 2.07 | 0.93 | 0.18 | 0.06 | 1.16 |
| *Juglans nigra* | 12.20 | 4.20 | 1.68 | 18.08 | 1.31 | 0.26 | 0.13 | 1.69 | 1.19 | 0.19 | 0.06 | 1.44 |
| *Juglans regia* | 12.08 | 4.24 | 1.69 | 18.01 | 1.33 | 0.26 | 0.12 | 1.70 | 1.11 | 0.18 | 0.04 | 1.33 |
| *Juglans sigillata* | 12.05 | 4.23 | 1.69 | 17.96 | 1.33 | 0.26 | 0.12 | 1.70 | 1.11 | 0.18 | 0.04 | 1.33 |
| *Juglans* average | 11.30 | 4.20 | 1.70 | 17.20 | 1.30 | 0.26 | 0.14 | 1.70 | 1.04 | 0.19 | 0.05 | 1.28 |
| *Platycarya strobilacea* | 4.06 | 5.34 | 2.43 | 11.83 | 0.36 | 0.34 | 0.21 | 0.92 | 0.38 | 0.28 | 0.31 | 0.96 |
| *Pterocarya fraxinifolia* | 12.24 | 4.20 | 1.64 | 18.08 | 1.36 | 0.24 | 0.14 | 1.74 | 1.16 | 0.19 | 0.08 | 1.43 |
| *Pterocarya hupehensis* | 12.16 | 4.33 | 1.68 | 18.17 | 1.38 | 0.26 | 0.14 | 1.78 | 1.04 | 0.21 | 0.09 | 1.34 |
| *Pterocarya macroptera var. insignis* | 12.13 | 4.15 | 1.63 | 17.90 | 1.34 | 0.24 | 0.14 | 1.73 | 1.14 | 0.20 | 0.06 | 1.39 |
| *Pterocarya stenoptera* | 4.06 | 4.18 | 1.68 | 9.91 | 0.44 | 0.24 | 0.13 | 0.81 | 0.34 | 0.18 | 0.06 | 0.58 |
| *Pterocarya tonkinensis* | 11.98 | 0.00 | 0.83 | 12.81 | 1.33 | 0.00 | 0.07 | 1.39 | 1.14 | 0.00 | 0.03 | 1.17 |
| average | 9.04 | 4.28 | 1.71 | 15.03 | 0.97 | 0.24 | 0.15 | 1.36 | 0.93 | 0.22 | 0.09 | 1.24 |

**Table S5c** Statistic of the detected indels across the *Carya*, *Engelhardia* and *Juglans* plastomes using *R. chiliantha* as outgroup.

| **Species** | **Feature** | **Deletion** | | **Insertion** | | **Percentage (%)** |
| --- | --- | --- | --- | --- | --- | --- |
|  |  | **No.** | **Total length (bp)** | **No.** | **Total length (bp)** |  |
| *Carya* | Intergenic regions | 1478 | 16850 | 1300 | 12947 | 49 |
|  | Exon | 192 | 2065 | 167 | 2931 | 6 |
|  | Intron | 1242 | 21404 | 1175 | 6941 | 43 |
|  | RNA Gene | 77 | 214 | 39 | 186 | 2 |
|  | Total | 2989 | 40533 | 2681 | 23005 | 100 |
| *Engelhardia* | Intergenic regions | 993 | 9455 | 1185 | 14314 | 55 |
|  | Exon | 183 | 2287 | 204 | 1680 | 10 |
|  | Intron | 702 | 7606 | 634 | 4638 | 33 |
|  | RNA Gene | 21 | 100 | 71 | 522 | 2 |
|  | Total | 1899 | 19448 | 2094 | 21154 | 100 |
| *Juglans* | Intergenic regions | 1613 | 17276 | 1258 | 12497 | 60 |
|  | Exon | 123 | 1217 | 133 | 2097 | 5 |
|  | Intron | 884 | 12001 | 573 | 2694 | 31 |
|  | RNA Gene | 102 | 282 | 78 | 329 | 4 |
|  | Total | 2722 | 30776 | 2042 | 17617 | 100 |

**Table S5d** Statistic of the detected indels across the fourteen-eight Juglandaceae plastomes using *R. chiliantha* as outgroup.

| **Feature** | **Deletion** | | **Insertion** | | **Percentage (%)** |
| --- | --- | --- | --- | --- | --- |
|  | **No.** | **Total length (bp)** | **No.** | **Total length (bp)** |  |
| Intergenic regions | 5643 | 59426 | 5282 | 54988 | 55 |
| Exon | 692 | 7842 | 723 | 9195 | 7 |
| Intron | 3819 | 52397 | 3223 | 19694 | 35 |
| RNA Gene | 274 | 821 | 274 | 1486 | 3 |
| Total | 10428 | 120486 | 9502 | 85363 | 100 |
