# Supplementary figures and images for: Plastome evolution of Engelhardia facilitates phylogeny of Juglandaceae
Source: BMC Plant Biol. 2024 Jul 6;24:634. doi: 10.1186/s12870-024-05293-0 (PMC11227234; doi:10.1186/s12870-024-05293-0)

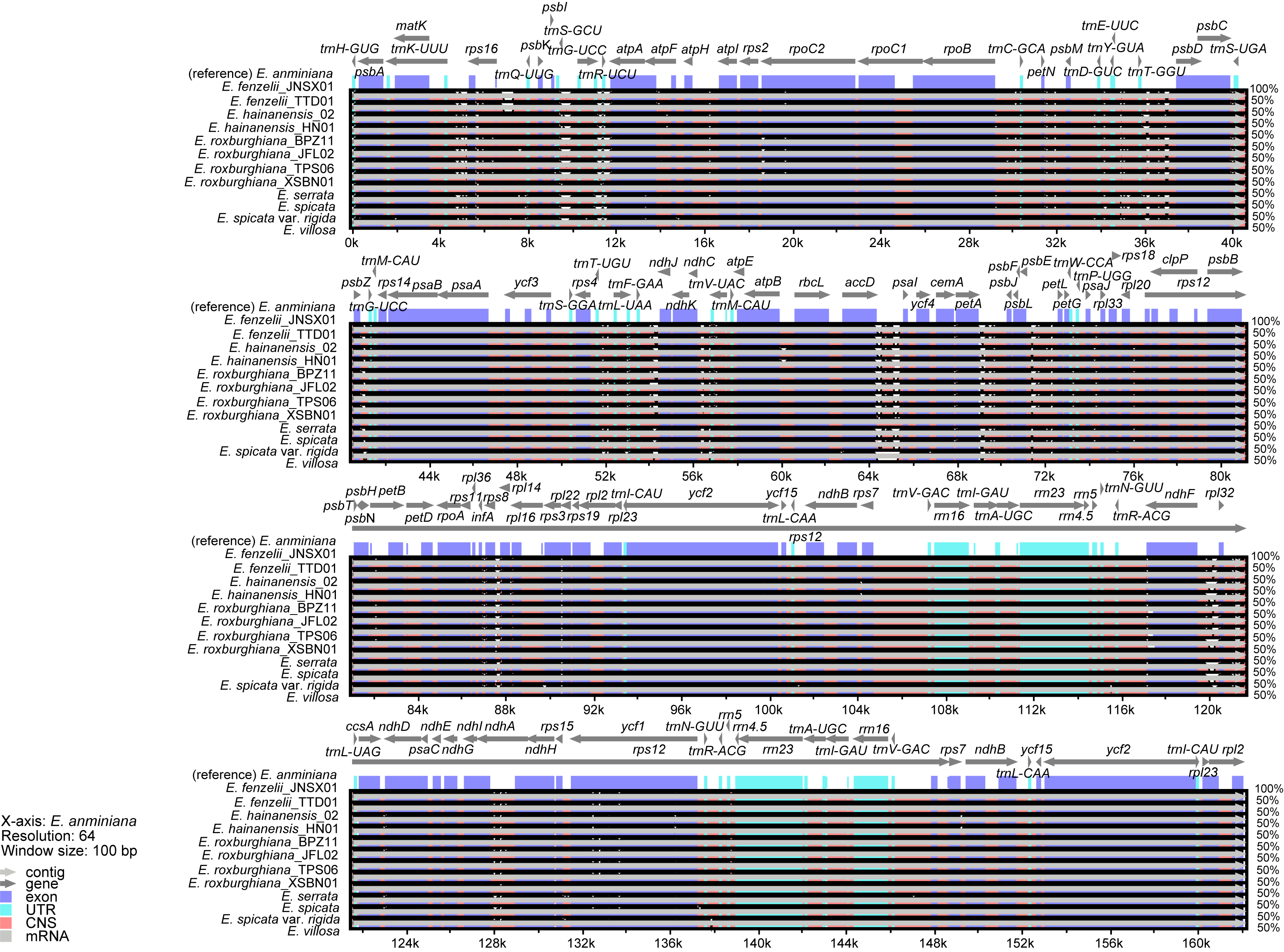

Supplement: Supplementary file 2 — Supplementary Material 2. [file 12870_2024_5293_MOESM2_ESM.zip › Supplementary figure/Figure S1.tif]

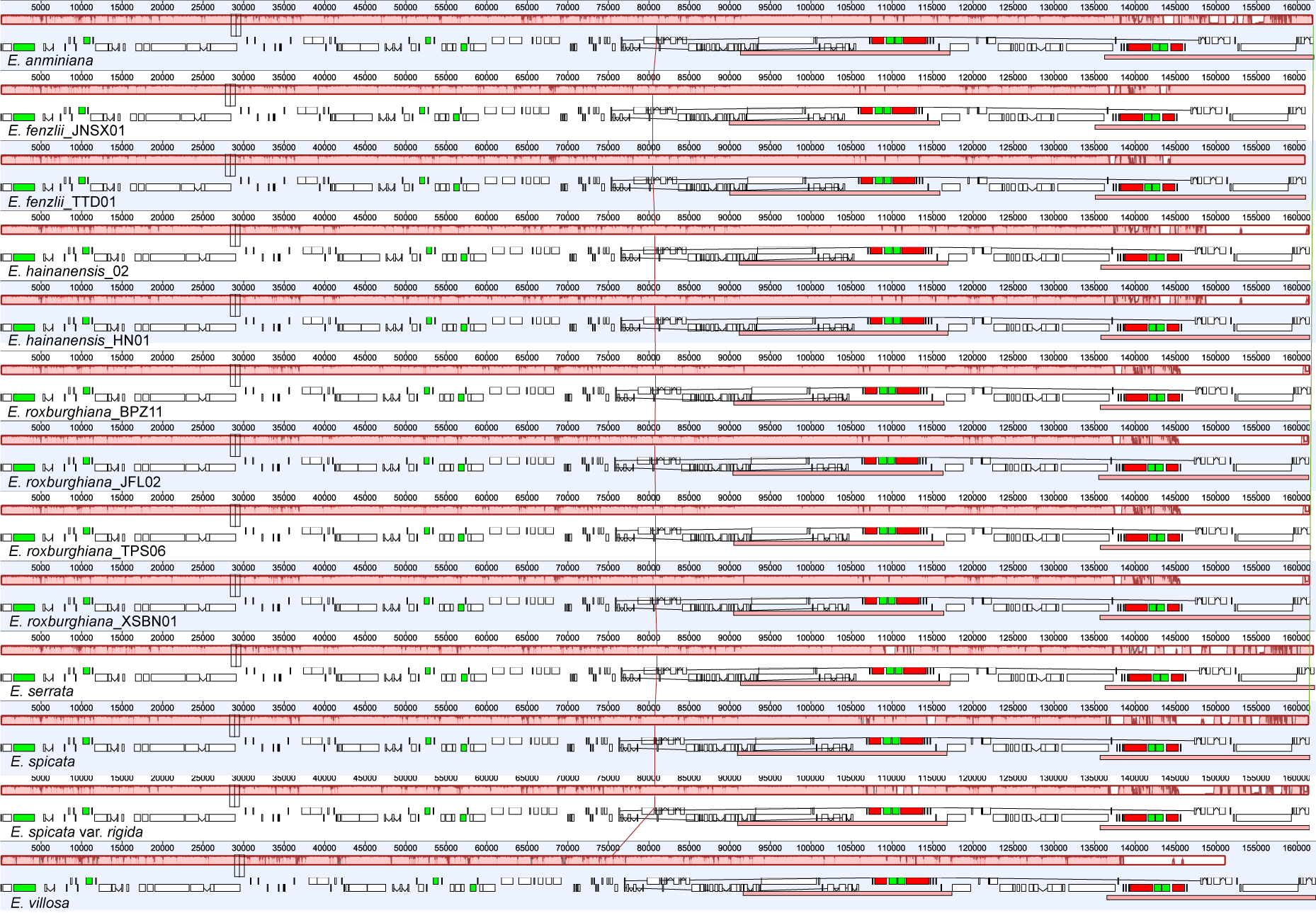

Supplement: Supplementary file 2 — Supplementary Material 2. [file 12870_2024_5293_MOESM2_ESM.zip › Supplementary figure/Figure S2.tif]

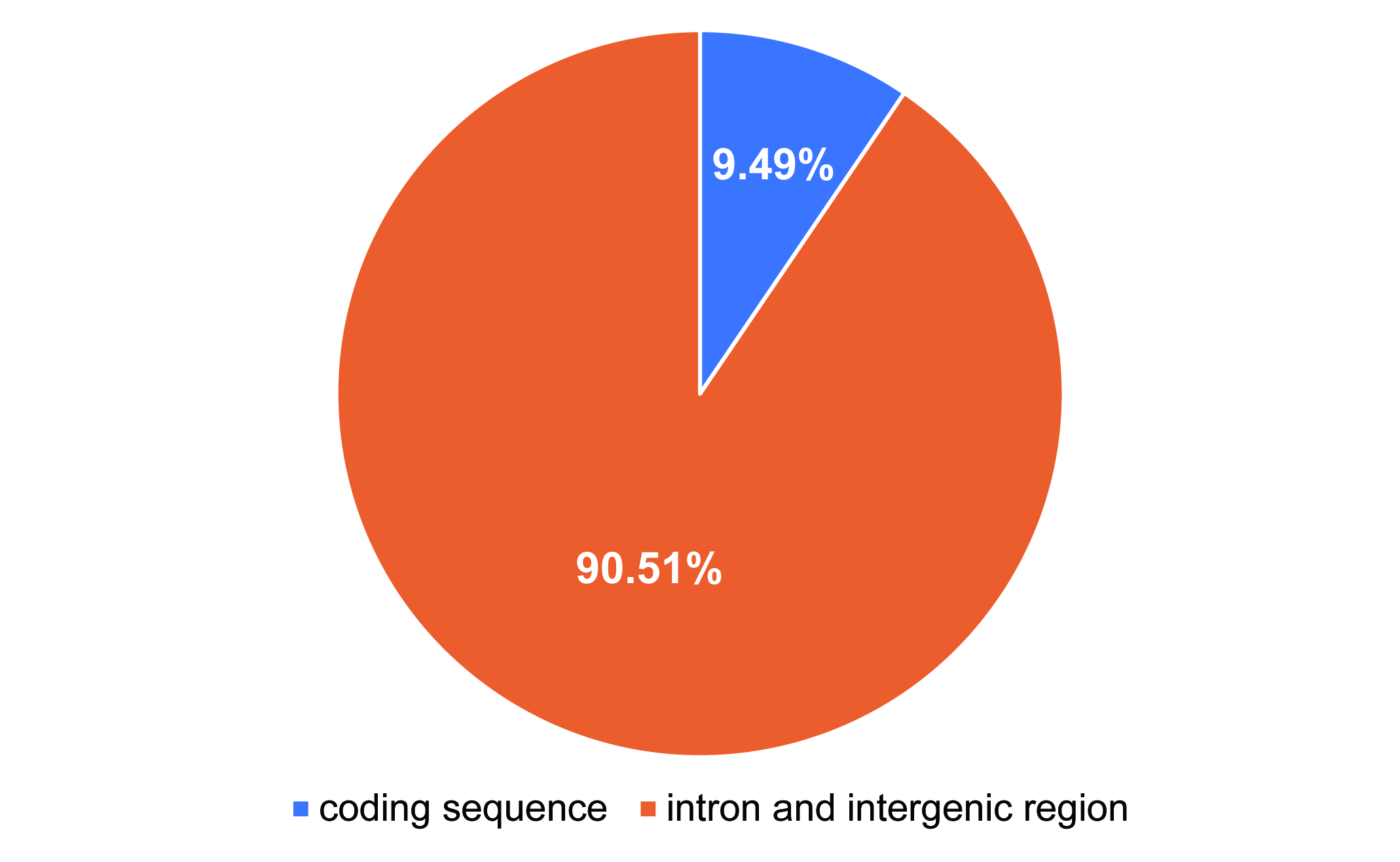

Supplement: Supplementary file 2 — Supplementary Material 2. [file 12870_2024_5293_MOESM2_ESM.zip › Supplementary figure/Figure S3.tif]

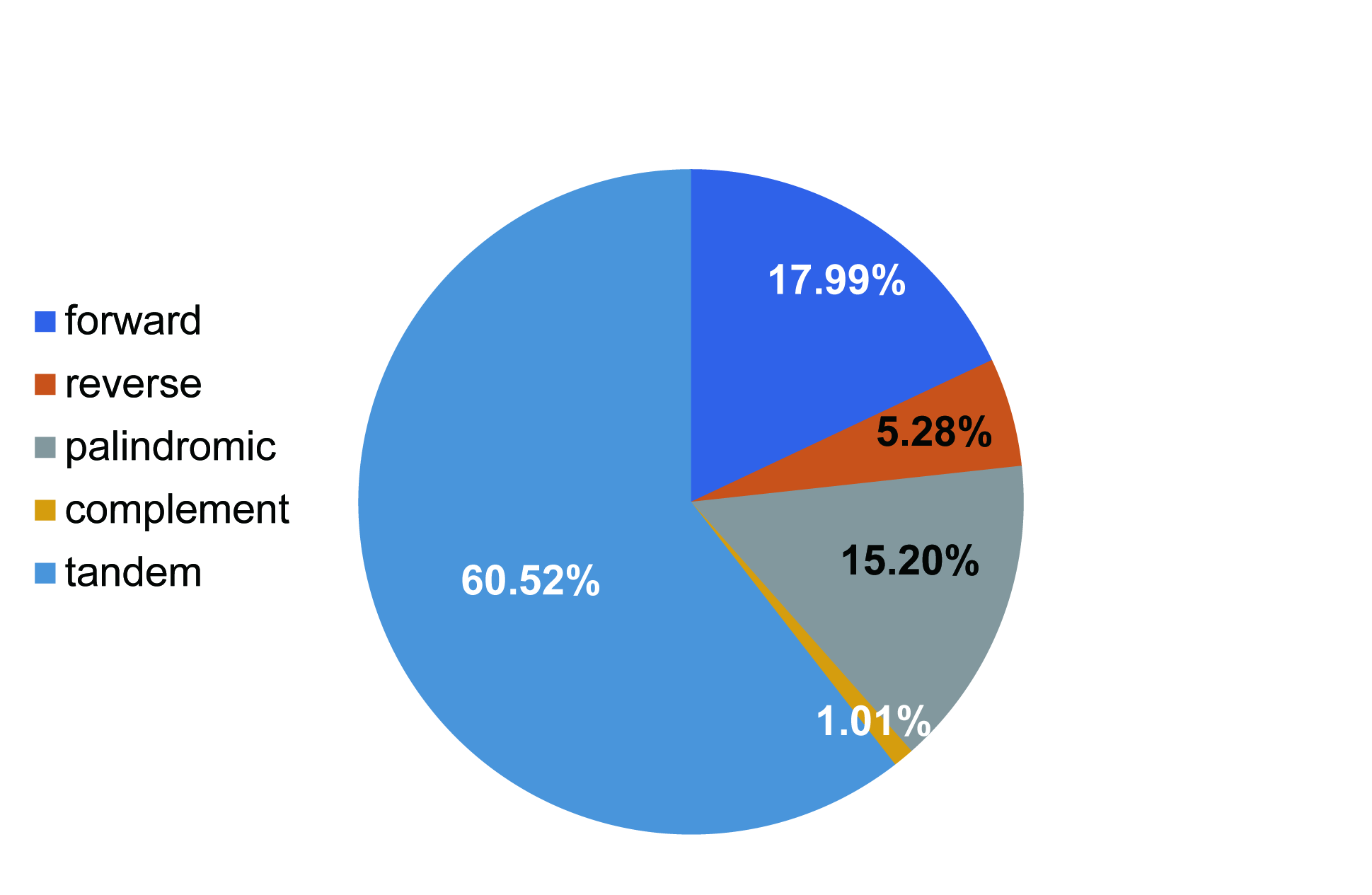

Supplement: Supplementary file 2 — Supplementary Material 2. [file 12870_2024_5293_MOESM2_ESM.zip › Supplementary figure/Figure S4.tif]

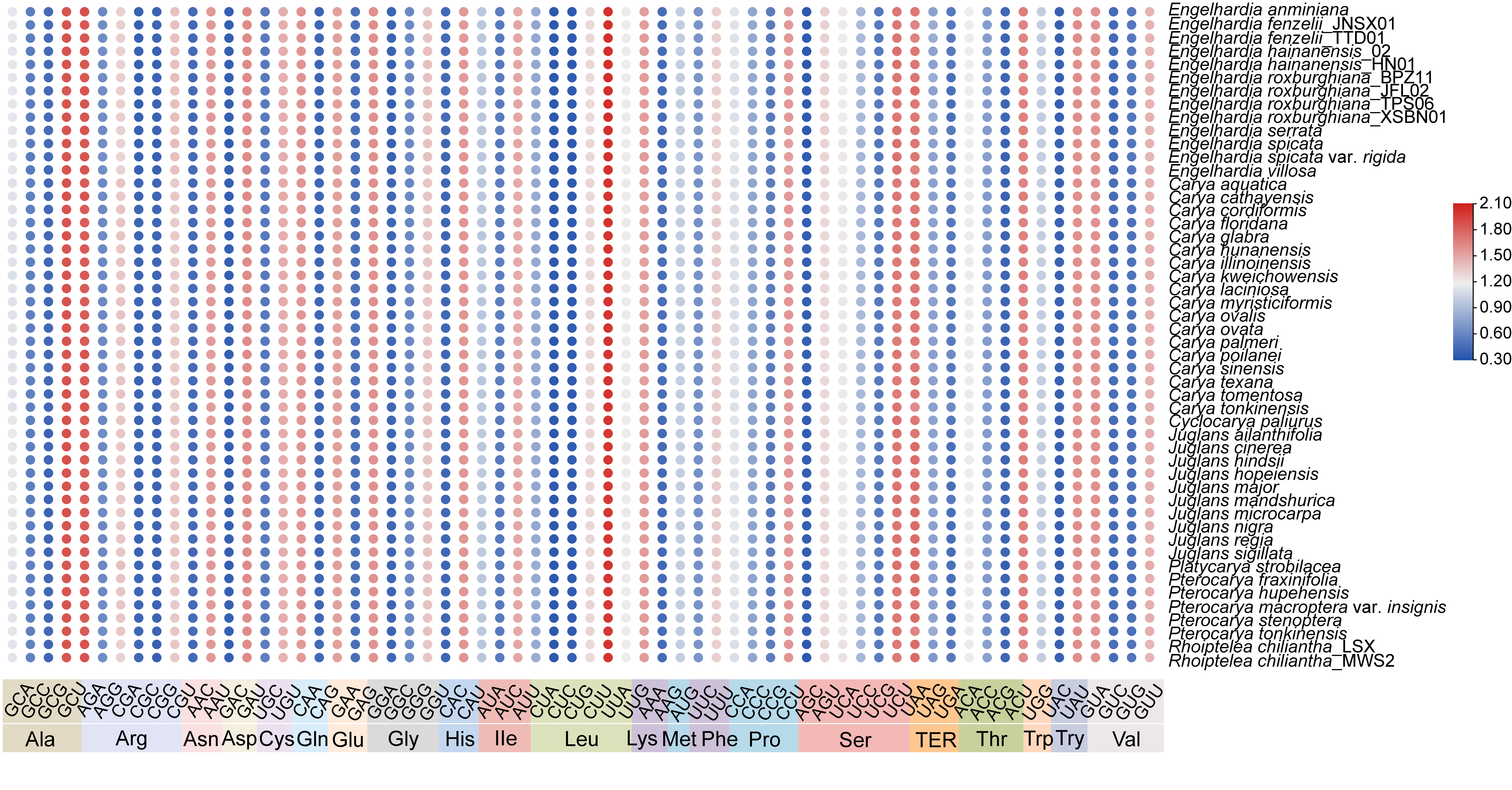

Supplement: Supplementary file 2 — Supplementary Material 2. [file 12870_2024_5293_MOESM2_ESM.zip › Supplementary figure/Figure S6.tif]
